# P.O.R.E.: A Programmable Ohmic Research Engine for Low-Cost Solid-State Nanopore Fabrication

**DOI:** 10.1109/access.2026.3727354

**Published:** 2026-08-26

**Authors:** CHAOMING GU, SANGWON LEE, KAMRUZZAMAN JOTY, NAVOD THYASHAN, MIN JUN KIM

**Affiliations:** 1 Department of Mechanical Engineering, Southern Methodist University, Dallas, TX 75205, USA; 2 School of Electrical Engineering, Hanyang University, ERICA Campus, Ansan-si, South Korea

**Keywords:** Solid-state nanopores, chemically-tuned controlled dielectric breakdown, low-cost fabrication, compact instrumentation

## Abstract

Solid-state nanopores have emerged as a versatile, label-free platform for single-molecule sensing. However, conventional *ex situ* fabrication techniques, such as focused ion beam milling and transmission electron microscopy drilling, are constrained by high cost, low throughput, and dependence on specialized instrumentation, limiting their widespread adoption and scalability. *In situ* fabrication based on controlled dielectric breakdown (CDB) greatly simplifies the process, yet existing implementations often remain expensive and rely on proprietary software, restricting flexibility for method development. Here, we present P.O.R.E., a low-cost, compact and programmable system for controlled dielectric breakdown nanopore formation. This platform achieves system-level miniaturization and cost reduction without sacrificing electrical precision. Its compact control circuit and modular hardware design enable convenient, high-fidelity interfacing with nanopore chips and peripheral components. A Python-based software interface provides real-time monitoring, programmable feedback control, and an observer-based scheme for real-time pore-size estimation, while offering extensibility for diverse fabrication protocols. The system is USB-powered and smartphone-sized, with the core electronic hardware costs less than 100 US dollars, significantly lowering the barrier to entry. Experimental validation demonstrates nanopore formation across the 6–20 nm range, with stable electrical characteristics and low-frequency noise. DNA and protein translocation measurements acquired using a dedicated low-noise amplifier further confirm the downstream sensing functionality of the fabricated pores. This work establishes an accessible and extensible platform for solid-state nanopore fabrication and provides a foundation for future expansion toward more automated and parallel fabrication workflows.

## I. INTRODUCTION

Solid-state nanopore, as a nanoscale aperture fabricated through various solid-state materials, has become a powerful platform for single-molecule analysis. It features label-free detection, controllability of pore geometry and physicochemical properties, flexible operating conditions, and compatibility with complex structures integration and multi-physics coupling [1], [2]. By employing solid-state nanopore devices, DNA [3], protein [4], nanoparticles [5], and their interaction dynamics [6] can be identified in real-time with single-molecular resolution.

The performance of solid-state nanopore devices is critically dependent on the precise control of pore geometry, which in turn is governed by the fabrication method. Early and still widely used approaches rely on *ex situ* milling, in which a focused beam of ions or high-energy electrons sculpts a pore directly through a freestanding dielectric membrane. Transmission electron microscopy (TEM) drilling and focused ion beam (FIB) milling can achieve sub-nanometer dimensional control and have produced some of the highest-quality pores reported to date [7], [8].

However, these techniques require access to costly and specialized instrumentation, demand highly trained operators, and extra approaches to improve the surface hydrophilicity of nanopores, resulting in low throughput and a high effective cost per device. These constraints fundamentally limit the scalability of solid-state nanopore technology and restrict its accessibility to a small number of well-equipped laboratories.

To overcome these barriers, *in situ* fabrication by controlled dielectric breakdown (CDB) has emerged as an attractive alternative. In CDB, a high electric field is applied across a thin dielectric membrane immersed in electrolyte, nucleating a single nanoscale pore at the point of dielectric breakdown [9]. Because CDB requires only a voltage source, a current measurement circuit, and a fluidic cell, it can be implemented at a small fraction of the cost of beam-based milling, and the chemically-tuned variant (CT-CDB) has further improved pore quality and stability [10].

Building on the available automated-CDB instrumentation and software pioneered by Waugh et al. [11], which reliably fabricates precisely sized, low-noise pores at a yield above 85 % but relies on a benchtop measurement setup, recent efforts have pushed toward compact, microcontroller-assisted platforms [12], [13]. Nuwan et al. [13] demonstrated an Arduino-based, USB-powered device costing under $150 that fabricated tailored pores in the 2.6 to 12.6 nm range with roughly 80 % of pores within 1 nm of the target diameter; however, its control application is built on the proprietary MATLAB environment, and its 58 mm × 107 mm board employs through-hole components that introduce parasitic effects and enlarge the footprint. Recently, concurrent with the present work, Cui et al. [12] reported a USB-powered device (170 mm × 100 mm × 55 mm, approximately 1 kg) that integrates a bipolar-voltage circuit with real-time Slope/Jerk breakdown detection and a dynamic PID size-control algorithm, achieving a size accuracy of ±0.5 nm over a 2 to 20 nm range. However, its customizable automation software is not released as a community-extensible framework, and its functional validation is confined to DNA.

To address these challenges, we present the Programmable Ohmic Research Engine (P.O.R.E.), a low-cost, compact, and extensible system for solid-state nanopore fabrication via CT-CDB. The contributions of this work are fourfold.

First, we design a compact control circuit based on a single Arduino microcontroller, a 12-bit DAC, a 16-bit ADC, and a three-stage operational-amplifier signal chain, achieving a current-measurement accuracy exceeding 99% while reducing the printed-circuit-board footprint by approximately 70% relative to prior work [13]. Second, we integrate all electrical, mechanical, and fluidic subsystems into a single USB-powered, smartphone-sized enclosure that requires no bench-top instrumentation. Third, we develop a Python/PyQt-based control software using an open-source software ecosystem, with a modular architecture that provides real-time monitoring, structured data logging, and an observer-based scheme for real-time pore-size estimation while facilitating extension to new fabrication protocols. Fourth, we experimentally validate the complete system by fabricating nanopores spanning 6~20 nm in diameter and demonstrate their electrical stability, low-noise characteristics, and single-molecule sensing capability for both DNA and protein analytes. The core-electronics cost remains below $100 USD, substantially lowering the barrier to entry for solid-state nanopore research.

The remainder of this paper is organized as follows. Section II describes the system architecture and circuit design; Section III details the control software design; Section IV presents the nanopore fabrication process together with pore-size estimation and post-fabrication electrical characterization; Section V validates the platform through single-molecule detection of DNA and protein; and Section VI concludes the paper.

## II. SYSTEM INTEGRATION AND CIRCUIT DESIGN

### A. SYSTEM ARCHITECTURE

**FIGURE 1.**
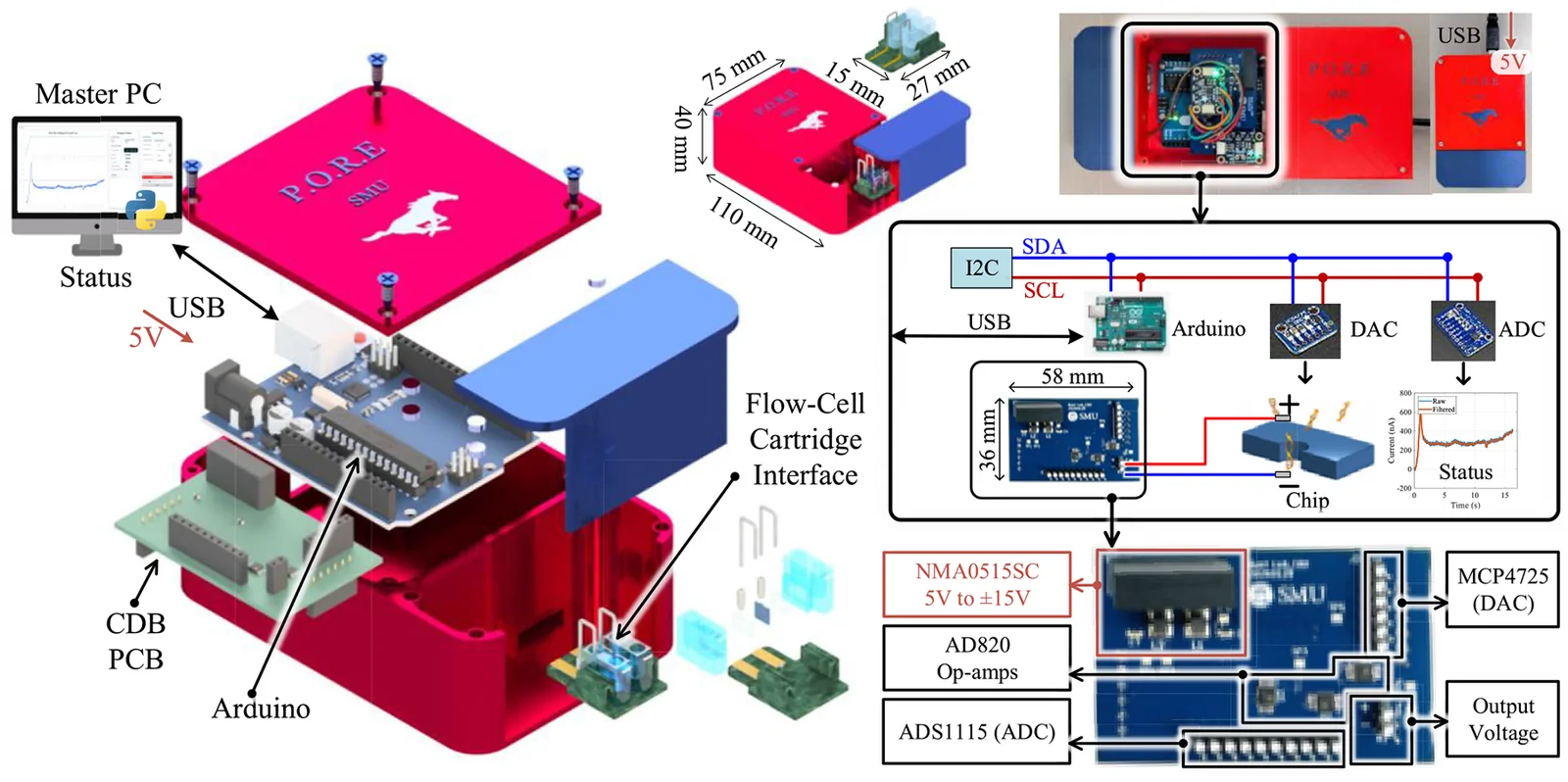
The P.O.R.E. system: an Arduino-based, USB-powered solid-state nanopore fabrication device featuring integrated electronics, a single-cable host interface, and a compact flow-cell cartridge design. The main PC transmits the voltage reference signal and receives the measured current for feedback-controlled pore fabrication. A DAC applies the reference voltage to the nanopore chip, while an ADC measures the pore current.

The P.O.R.E. system integrates the electronic, mechanical, and fluidic subsystems required for nanopore fabrication into a single self-contained platform powered entirely through a standard USB connection to the host computer. Consequently, no external power supply, signal generator, or additional instrumentation is required. As shown in Fig. 1, the assembled device measures 110 mm × 75 mm × 40 mm, comparable in size to a consumer smartphone, and becomes fully operational after connecting a single USB cable. This compact form-factor distinguishes the system from conventional nanopore fabrication setups that typically require multiple benchtop instruments and complex wiring configurations.

#### Housing and Lid Design.

The enclosure is fabricated using fused deposition modeling (FDM) 3D printing [14] and consists of three mechanically distinct sections: a primary housing box that encloses the control electronics, and a magnetically retained removable lid. As shown in Fig. 1 left, opening the fastened lid (red) exposes the internal electronics. The fixed lid on the top surface is secured by four M4 × 0.7 screws, protects the electronics under operating conditions. The magnetic lid closure (blue), implemented with permanent magnets, eliminates fastener requirements for chip loading, provides a repeatable seating position on each re-engagement.

#### Internal Electronics.

Within the enclosure, the Arduino UNO R3 and the CDB PCB are arranged in a vertically stacked configuration to maximize component density. The Arduino occupies the lower position and interfaces with the host computer through a single USB connection, which provides 5 V power and bidirectional UART communication. The PCB is mounted above the microcontroller and routes analog signals to the flow-cell cartridge through dedicated cartridge connectors integrated into the housing. This arrangement contributes to the compact footprint while maintaining straightforward assembly and accessibility.

#### Flow-Cell Cartridge Interface.

The flow-cell cartridge, measuring 27 mm × 15 mm, houses the nanopore chip between male and female flow-cell halves. Polydimethylsiloxane (PDMS) gaskets positioned on both the cis and trans sides of the chip provide a leak-tight seal without requiring adhesive bonding. Electrical contact with the electrolyte is provided by Ag/AgCl wire electrodes inserted through cartridge-integrated electrode pins. Owing to their stable half-cell potential and minimal polarization effects, Ag/AgCl electrodes are well suited for nanopore fabrication and ionic current measurements [13]. The signal path between the nanopore chip and the control electronics are routed through the cartridge connectors.

#### Host Interface.

A Python-based graphical user interface running on the host PC provides real-time monitoring of current and voltage signals, fabrication parameter control, and data logging functionality. Communication between the software and hardware is achieved entirely through the same USB connection used for power delivery, reducing the system interface to a single cable.

The electrical architecture of the proposed nanopore fabrication system follows an architecture similar to those reported in [11], [12], and [13] as illustrated in Fig. 1 right. The host PC generates a voltage reference signal and transmits it to an Arduino UNO R3 operating at a 16 MHz clock frequency via UART communication at 115,200 bps. A 12-bit digital-to-analog converter (DAC), MCP4725, converts the received digital command into an analog voltage with an output range of 0–3.3 V and a voltage resolution of 0.805 mV/LSB. The generated signal is subsequently amplified by operational amplifiers and applied to the nanopore chip.

The resulting pore current is digitized using an ADS1115 analog-to-digital converter (ADC) configured with a full-scale input range of ±4.096 V and a voltage resolution of 125μV/LSB. The ADC operates at its maximum sampling rate of 860 samples/s, and the acquired measurements are transmitted back to the host PC through UART communication for feedback-controlled pore fabrication. Both the DAC and ADC communicate with the Arduino through an I2C interface operating at 400 kHz.

The custom CDB PCB used for nanopore fabrication measures 58 mm × 36 mm, as shown in Fig. 1. Compared with the PCB reported in [13] (58 mm × 107 mm), the proposed design achieves approximately 70% size reduction while significantly increasing circuit density. Unlike the previous design, which utilized through-hole resistors, capacitors, and operational amplifiers, all components in the proposed PCB are implemented using surface-mounted device (SMD) packages.

This approach reduces parasitic capacitance, inductance, and resistance while enabling substantial board miniaturization. Furthermore, the dual power supply architecture reported in [13] is replaced by a compact MURATA NMA0515SC DC–DC converter that generates ±15 V from a 5 V input, allowing all electronic subsystems to be integrated onto a single PCB. The 5 V input is provided by the host PC via the USB interface, as depicted in Fig. 1.

The cost breakdown of the PCB components is summarized in Table 1. The Arduino UNO R3 represents the highest-cost component at $ 27.60, while the total hardware cost of the proposed system is $ 85.72, approximately $ 50 lower than the cost reported in [13]. This reduction is achieved through PCB miniaturization, adoption of SMD components, and simplification of the power supply architecture.

The reported cost of USD 85.72 represents the core electronic hardware, including the Arduino microcontroller, custom PCB, ADC, DAC, operational amplifiers, power converter, passive components, and PCB fabrication. The reusable enclosure and flow-cell cartridge, as well as experimental consumables such as nanopore chips, PDMS gaskets, Ag/AgCl electrodes, electrolyte, and connection cables, are not included because their costs depend on fabrication method, supplier, and reuse conditions.

The resulting architecture provides a portable and low-cost nanopore fabrication platform with a core-electronics cost below $100 USD. Despite its compact footprint, the system delivers integrated nanopore fabrication, current monitoring, and feedback-control capabilities, making advanced nanopore research accessible to laboratories without specialized instrumentation infrastructure.

### B. CIRCUIT DESIGN

**FIGURE 2.**
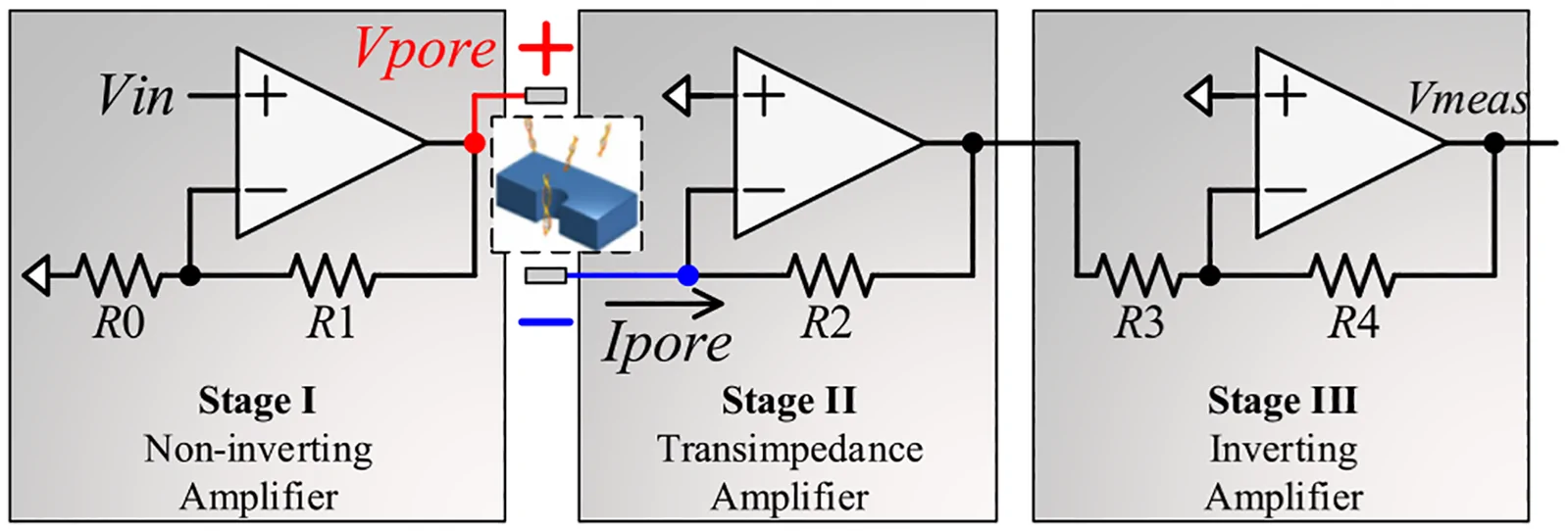
Circuit design for voltage application and current measurement in nanopore fabrication. Three operational amplifiers are configured in three stages: Stage I as a non-inverting amplifier, Stage II as a transimpedance amplifier, and Stage III as an inverting amplifier.

The pore voltage and current are controlled and measured using the AD820 operational amplifier with a unity-gain bandwidth of 1.8 MHz, a slew rate of 3V/μs, and dual-supply capability up to ±15 V. As shown in Fig. 2, three AD820 operational amplifiers are configured in three stages. Stage I uses a non-inverting amplifier topology, where the DAC output voltage $V_{\text{in}}$ is amplified to $V_{\text{pore}}$ according to (1) for pore fabrication on the chip.

(1) $$V_{\text{pore}}=\left(\frac{R1}{R0}+1\right)V_{\text{in}}$$

where R0=10kΩ and R1=49.9kΩ are feedback gain resistors shown in Fig. 2, resulting in a voltage gain of 5.99. As $V_{\text{in}}$ varies from 0 to 2.5 V, $V_{\text{pore}}$ varies from 0 to 15 V, providing a sufficient voltage level for nanopore fabrication.

$V_{\text{pore}}$ is applied to the positive electrode with respect to the virtual ground connected to the negative electrode, as illustrated in Fig. 2. The pore current $I_{\text{pore}}$ flowing through the chip is then given by:

(2) $$I_{\text{pore}}=\frac{V_{\text{pore}}}{Z_{\text{pore}}}$$

where $Z_{\text{pore}}$ is the impedance of the chip.

The transimpedance and inverting amplifiers in Stages II and III then convert $I_{\text{pore}}$ into the corresponding measured voltage $V_{\text{meas}}$ as:

(3) $$V_{\text{meas}}=\left(\frac{R2\times R4}{R3}\right)I_{\text{pore}}$$

where R2=1MΩ,R3=10kΩ and R4=100kΩ are feedback gain resistors depicted in Fig. 2, resulting in a current-to-voltage gain of 1 × 10^7^ V/A.

Finally, the measured pore current $I_{\text{pore}}$ is computed by rearranging (3) as follows:

(4) $$I_{\text{pore}}=V_{\text{meas}}\left(\frac{R3}{R2\times R4}\right)$$

### C. SYSTEM PERFORMANCE

The amplification gain in (3) was designed to be 1 × 10^7^ V/A using R2,R3 and R4. Subsequently, the amplified voltage is attenuated by a factor of 4 via a resistive voltage divider prior to digitized by the ADC. Therefore, the effective gain is 2.5 × 10^6^ V/A, yielding a ±1.6384μA linear measurement range and a theoretical current resolution of 0.05 nA/LSB.

**FIGURE 3.**
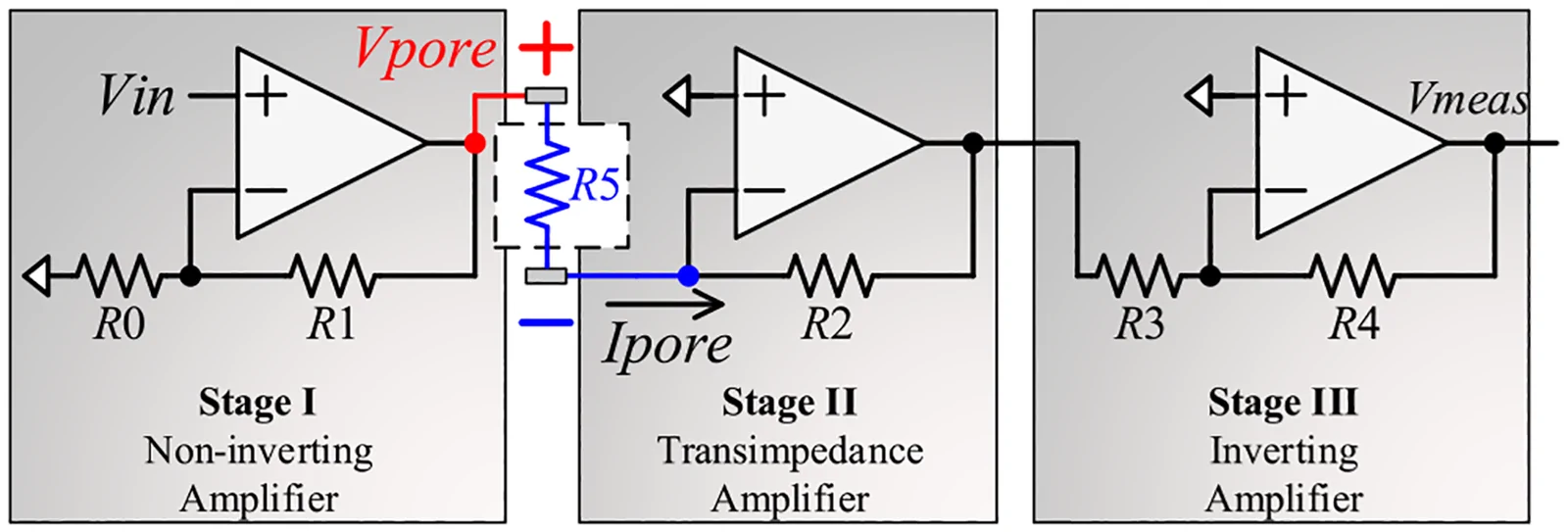
Replacement of the chip with resistive loads R5 for current measurement accuracy testing.

**FIGURE 4.**
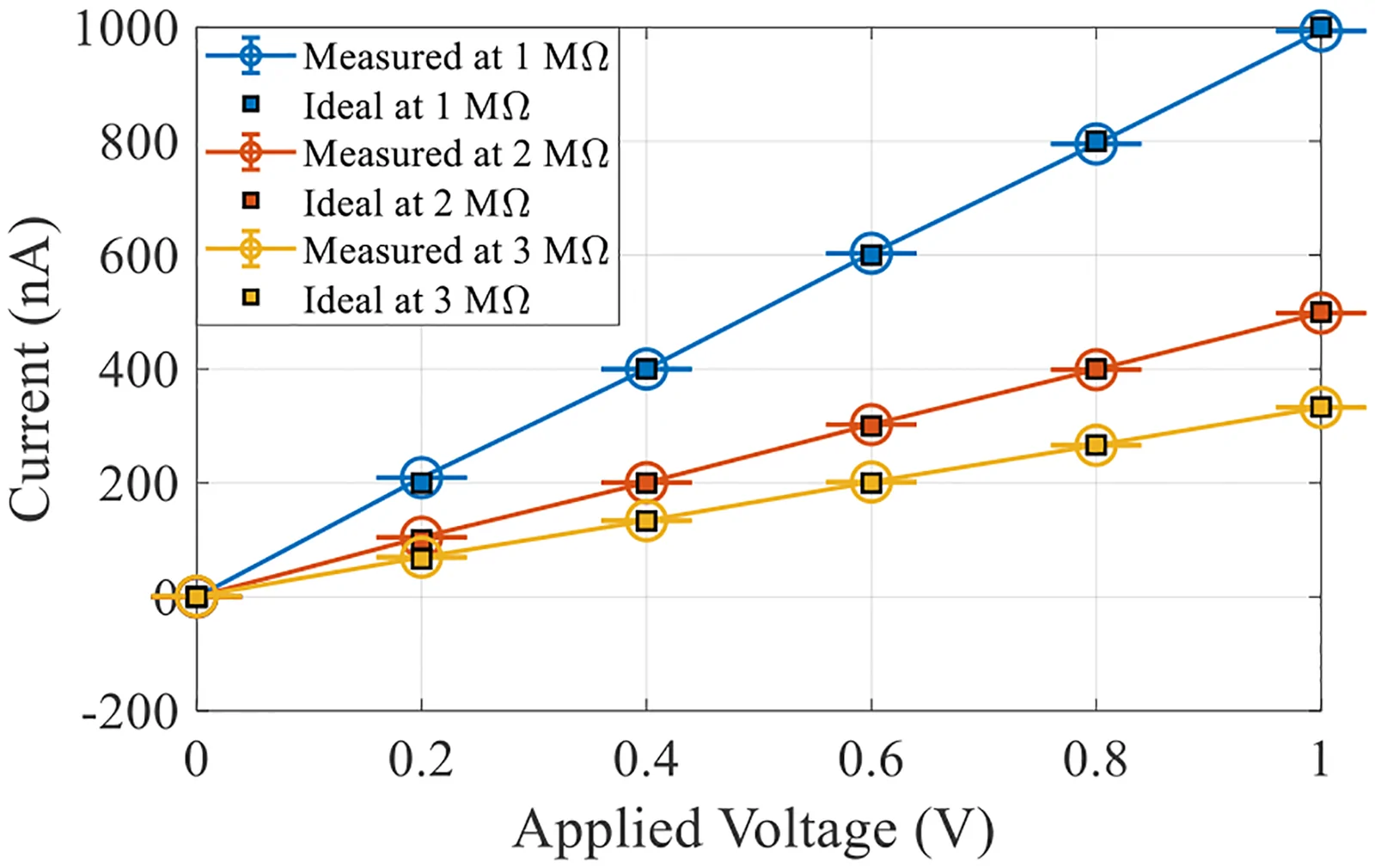
Current measurement accuracy for different R5 values of 1MΩ,2MΩ, and 3MΩ.

The measurement accuracy of the system was validated using different resistive loads, denoted as *R*5 in Fig. 3. The tested load conditions were R5=1MΩ,2MΩ,3MΩ, and experimental results in Fig. 4 demonstrated that the measurement accuracy exceeded 99% for all tested resistive loads, as evaluated using (5).

(5) $$\text{Accuracy}=\left(1-\left|\frac{I_{\text{pore}}^{avg}-I_{\text{pore}}^{*}}{I_{\text{pore}}^{*}}\right|\right)\times 100$$

where $I_{\text{pore}}^{*}$ is the theoretical (ideal) value for a given resistive load and applied voltage level, while $I_{\text{pore}}^{\text{avg}}$ is the average $I_{\text{pore}}$ value calculated from 100 samples collected at each voltage level.

The coefficient of determination R^2^ was also evaluated using (6). For all tested resistive loads, the obtained R^2^ values were more than 0.9996, indicating excellent agreement between the measured and ideal results.

(6) $${\text{R}}^{2}=\left(1-\frac{\sum_{i=1}^{N}\;{\left(I_{\text{pore},i}^{*}-I_{\text{pore},i}^{\text{avg}}\right)}^{2}}{\sum_{i=1}^{N}\;{\left(I_{\text{pore},i}^{*}-I_{\text{pore}}^{*,\text{avg}\;}\right)}^{2}}\right)$$

where the subscript i denotes each voltage level, and N=6 is the number of voltage levels.

## III. CONTROL SOFTWARE DESIGN

**FIGURE 5.**
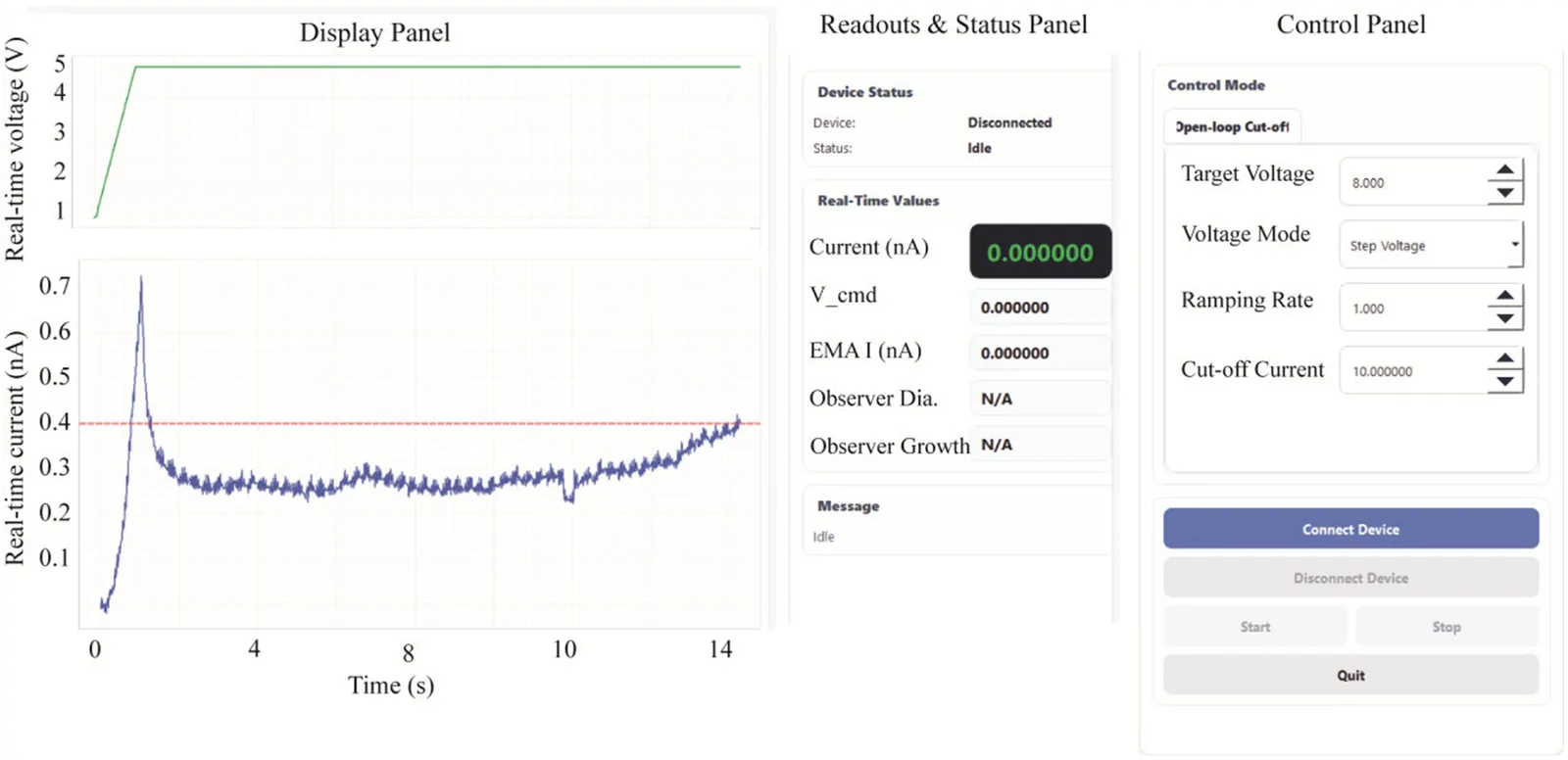
P.O.R.E. Controller overview: the graphical user interface (GUI) for P.O.R.E. system. GUI displays current and voltage readouts in both graph and numbers, with control panel for fabrication parameters settings. The display panel exhibits real voltage and current curve during the fabrication, in which green curve indicates real-time voltage (ramping mode), red line for cut-off threshold and blue curve for real-time current. Critical parameters are enlarged for better view.

To control the fabrication process through P.O.R.E. hardware in an intuitive manner, we designed a system-compatible graphical user interface (GUI) with rich functionality and high extensibility (Fig. 5). So far, related work [13] heavily relies on non-free proprietary software such as MATLAB to build their control applications, which increases the expense, increase the barrier to adoption, and limits the scalability. To further reduce software cost and facilitate customization, the control application was developed using the Python/PyQt ecosystem rather than proprietary commercial software.

The P.O.R.E. controller was implemented in Python 3.10.20 using PyQt 5.15 for the graphical user interface. Communication between the host application and the Arduino UNO R3 is performed through USB serial communication at 115,200 bps. At the microcontroller level, the MCP4725 DAC and ADS1115 ADC communicate with the Arduino through a 400-kHz I2C bus. The Arduino firmware is responsible for receiving voltage commands, updating the DAC output, acquiring ADC measurements, and returning electrical readbacks, whereas experimental sequencing, fabrication-recipe execution, threshold evaluation, visualization, and data logging are handled by the host-side Python application.

**FIGURE 6.**
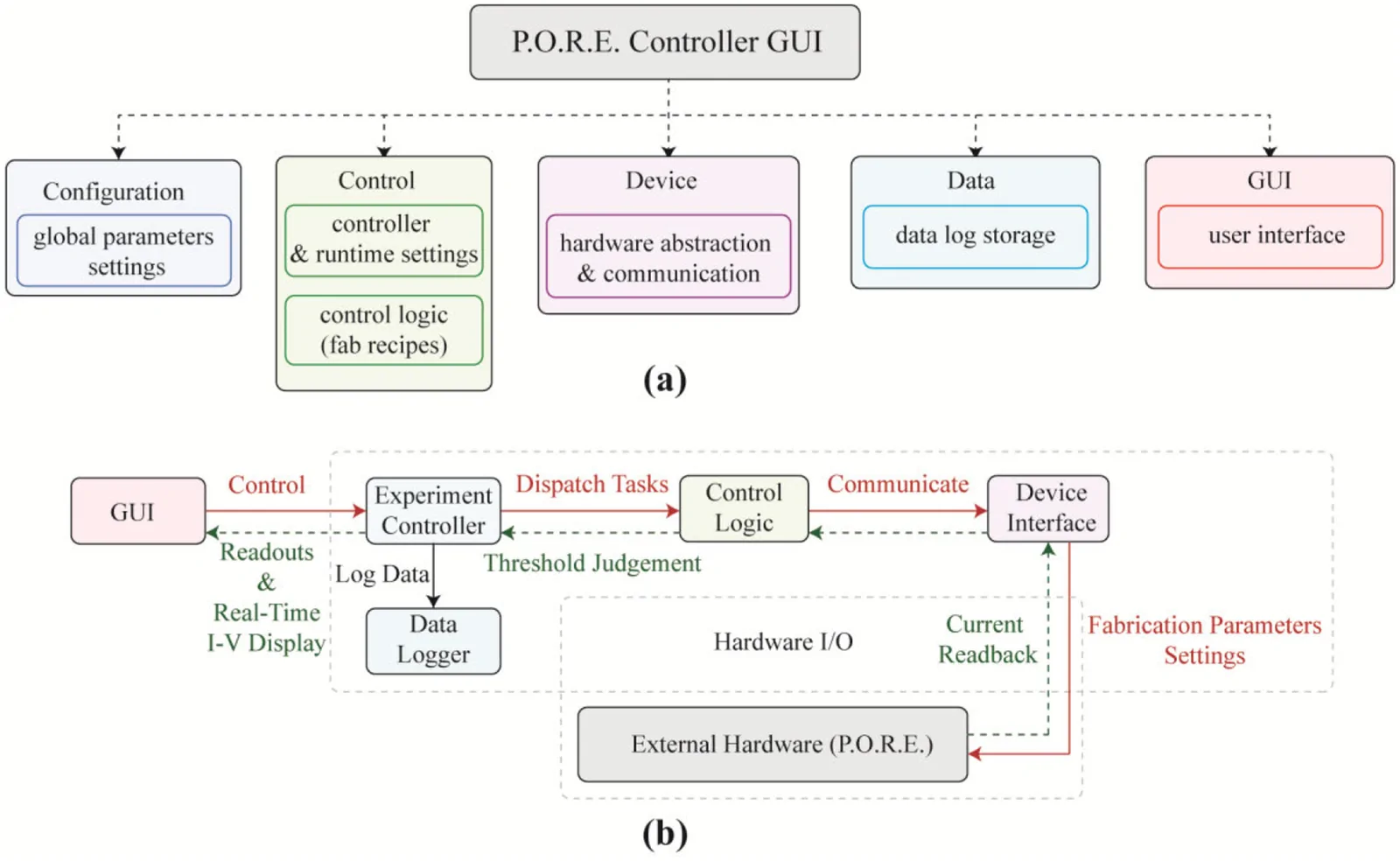
(a) The structure components of P.O.R.E. controller; (b) The basic interface logic of P.O.R.E. controller between GUI and hardware device.

The software serves as the central hub through which users configure experimental parameters, execute fabrication recipes, monitor real-time electrical readbacks, and log experimental data for post-process analysis. The overall architecture, illustrated in Fig. 6 (a), adopts a modular design philosophy that separates concerns into five distinct packages: Configuration, Control, Device, Data, and GUI. This separation enhances maintainability, facilitates independent testing of each module, and allows future extension without requiring modifications to unrelated components.

The Configuration module manages all global parameter settings that govern the behavior of the system and determine the fundamental hardware set-up. These include user-defined fabrication default values, hardware baud rate, communication port assignments, and voltage/current gain calibrations. By centralizing configuration data, the system ensures consistency across all modules and enables rapid reconfiguration between experimental runs without modifying source code.

The Control module constitutes the core logic of the application and is further divided into two sub-components. The first, the controller and runtime settings manager, orchestrates the overall experimental workflow by coordinating the sequencing of fabrication steps, managing state transitions, performing DC offset correction, and enforcing runtime constraints within multi-thread architecture. The second sub-component encapsulates the control logic in the form of fabrication recipes. Rather than simple parameter sets, these recipes implement complete fabrication workflows comprising sequential voltage operations, runtime state transitions, conditional threshold evaluation, and termination logic for specific fabrication protocols. This recipe-based architecture allows users to define, store, and reuse complex fabrication procedures, thereby improving experimental reproducibility. We have integrated commonly-used CT-CDB protocol which controls the fabrication process by monitoring leakage current and adjusting cut-off thresholds in real-time.

The Device module provides a hardware abstraction and communication layer that decouples the control logic from the specifics of the underlying instrumentation. By exposing a unified interface for sending commands and receiving readback data, this module enables the software to support different hardware configurations or communication protocols (e.g., serial and ethernet) with minimal changes to the higher-level control logic.

The Data module is responsible for data log storage. It captures all relevant experimental data at an 80 Hz sampling rate in csv structural format, including applied voltage, measured current, and controller status. Each experimental record is associated with a host-generated date/time stamp using the system clock in the format YYYYMMDD_HHMMSS. The timestamp therefore provides second-level identification of individual experimental runs, while the temporal evolution within each run is determined by the 80-Hz logging interval. This module ensures that every fabrication run is fully documented, supporting trace-ability and facilitating statistical analysis across multiple experiments.

The GUI module implements the user interface layer with three panels: applied voltage and current readback monitors in real-time, data readouts and system status display, and fabrication parameter controls, providing users with interactive controls for parameter entry, recipe selection, and experiment initiation, as well as real-time visualization of electrical characteristics during fabrication.

Fig. 6 (b) illustrates the runtime interaction among the software components during a typical fabrication experiment. The user initiates an experiment through the GUI, which issues control commands to the Experiment Controller. Upon receiving these commands, the Experiment Controller dispatches specific tasks to the Control Logic engine according to the selected fabrication recipe. The Control Logic module translates these high-level tasks into hardware-level instructions and communicates them to the Device Interface, which in turn transmits the critical fabrication parameter settings (i.e. voltage command) to the external P.O.R.E. hardware.

During fabrication, the external hardware continuously reports current readback values to the Device Interface via the hardware I/O channel. These readback signals propagate back through the Control Logic module, where they are evaluated against predefined threshold conditions, a process referred to as threshold judgment. This closed-loop mechanism enables the system to make autonomous decisions during fabrication, such as terminating a fabrication run upon exceeding the cut-off current threshold.

Simultaneously, the Experiment Controller forwards all acquired data to the Data Logger for persistent storage. The GUI receives readout values and renders them as real-time voltage and current curves, providing the operator with immediate visual feedback on the progress and outcome of the fabrication process. This real-time display is essential for monitoring device behavior during the fabrication process and for enabling manual intervention when necessary.

Several design principles guided the development of the software. First, the strict separation between control logic and hardware communication ensures portability: the same fabrication recipes can be executed on different hardware setups by substituting only the Device module. Second, the recipe-based control architecture promotes reproducibility, as identical fabrication protocols can be reliably replicated across sessions and across different users. Third, the integrated data logging and real-time visualization capabilities support a closed-loop experimental workflow, in which users can iteratively refine fabrication parameters based on immediate feedback. Finally, the modular structure facilitates extensibility, allowing new features to be incorporated with minimal impact on the existing codebase, such as additional analysis routines, alternative control algorithms, or support for other emerging instrumentations.

For safety, the high-voltage rails are galvanically isolated up to 1.5 kV DC from the host PC via a DC-DC converter (MURATA NMA0515SC). The system draws less than 200 mA (<1 W total power), maintaining a negligible thermal rise under 4 °C. Crucially, the Arduino firmware provides an autonomous fail-safe that immediately commands the voltage to 0 V and grounds the flow cell if the host GUI freezes or the serial connection drops. Under normal host-controlled threshold operation, the complete software feedback loop is updated at 80 Hz, corresponding to an interval of approximately 12.5 ms.

## IV. NANOPORE FABRICATION

### A. BUFFER AND ELECTRODE PREPARATION

A 45 mL stock solution of 1 M KCl and 10 mM Tris at pH 7.6 was prepared by dissolving 3.355 g of KCl (P9333, Sigma-Aldrich) and adding 450μL of 1 M Tris buffer at pH 7.6 (J61036, Alfa Aesar). Filtered deionized water was added to obtain a final volume of 45 mL and the final pH was set to pH 7.6 by adding drops of 1 M HCl or 1 M KOH. Hereafter, this solution is referred to as 1 M KCl-Tris.

Ag/AgCl electrodes were prepared by immersing Ag wires in a sodium hypochlorite solution overnight. The prepared electrodes were used for both nanopore fabrication and sensing experiments.

### B. FLOW CELL PREPARATION AND CHEMICALLY-TUNED CONTROLLED DIELECTRIC BREAKDOWN

The P.O.R.E. system was evaluated throughout the CT-CDB fabrication of solid-state nanopores with various pore sizes using the P.O.R.E. Controller graphical user interface (GUI), as shown in Fig. 5. The chip and flow-cell preparation procedures were adapted from the established CT-CDB protocol [10].

Each 4 mm × 4 mm chip consisted of a 12 ± 2 nm thick, low stress SiN_x_ membrane deposited on a 300μm thick Si substrate. A 10μm×10μm window was etched at the center of the chip to form a freestanding SiN_x_ membrane. Before flow cell assembly, the chips were rinsed with ethanol and dried using nitrogen gas. Care was taken during nitrogen drying to avoid damaging the freestanding membrane.

Through-holes were punched in two PDMS layers. The chip was placed between these layers such that the freestanding membrane window was aligned with the PDMS through holes. The PDMS-chip assembly was then secured between two flow cell components to form the complete flow cell assembly. To improve the hydrophilicity and wetting of the flow cell channels, both chambers were filled with ethanol and placed under vacuum in a desiccator.

Before nanopore fabrication, the assembled flow cell was rinsed twice with filtered deionized water and once with 1 M KCl-Tris. Finally, both reservoirs were filled with a mixture of 1 M KCl-Tris and NaOCl solution (425044, Sigma-Aldrich) with 10–15% available chlorine at a volume ratio of 9:2.

The electrolyte, KCl provides ionic conductivity required for current monitoring, while Tris buffer maintains a stable chemical environment during fabrication. NaOCl acts as the reactive oxidizing component. Under an applied transmembrane voltage, defect accumulation weakens Si-N bonds within the SiN_x_ membrane. OCl^−^ species then oxidizes the exposed, silicon-rich sites near the defects, converting the local SiN_x_ surface into a more oxygen-rich layer of hydrophilic silanol groups. The above oxidative modification proceeds concurrently from both membrane-electrolyte interfaces toward the high-field defect region. The resulting symmetric chemical boundary conditions limit preferential erosion from either surface, allowing the two oxidation fronts to converge through the membrane thickness. This balanced bilateral process is understood to suppress one-sided widening that would otherwise produce a tapered or conical pore and instead favors a more symmetric pore profile. This mechanism also produces a hydrophilic, oxygen-rich pore surface associated with Ohmic conductance, reduced low-frequency noise, and improved resistance to analyte adsorption and clogging [10], [15], [16].

As described in previous section, the system operates in a current cut-off control mode, in which the transmembrane ionic current is monitored continuously throughout the fabrication process. The user specifies the critical control parameters on the control panel, such as target voltage and cut-off current threshold prior to the experiment (Table 2). Since the quality of membrane varies depending on the number and distribution of defects, the target voltage is usually initiated from lower value such as 4 V to avoid membrane fracture. Then the voltage could be increased gradually. From our experiments, the typical breakdown voltage is in the range of 7~9 V. When the measured current exceeds the cut-off threshold, the system automatically removes the applied voltage to prevent further pore growth. Because the ionic current through a nanopore is directly related to the pore geometry through the conductance-based model [17], selecting an appropriate cut-off current effectively targets a specific pore diameter. In general, when a smaller pore size is needed (<10 nm), the threshold can be set to 1.2~1.5 times real-time leakage current; to obtain larger pores (>10 nm), 2× threshold or even higher level can be applied. This level of cut-off should be adjusted dynamically during the process since the leakage current level may fluctuate before the breakdown. This feedback-driven voltage auto-termination eliminates the need for manual intervention during the critical moment of dielectric breakdown, thereby reducing the risk of uncontrolled pore enlargement that commonly arises from the inherent latency of human response.

### C. VOLTAGE APPLICATION PROFILES

The P.O.R.E. system supports two distinct voltage application profiles for CT-CDB fabrication, each suited to different experimental requirements.

**FIGURE 7.**
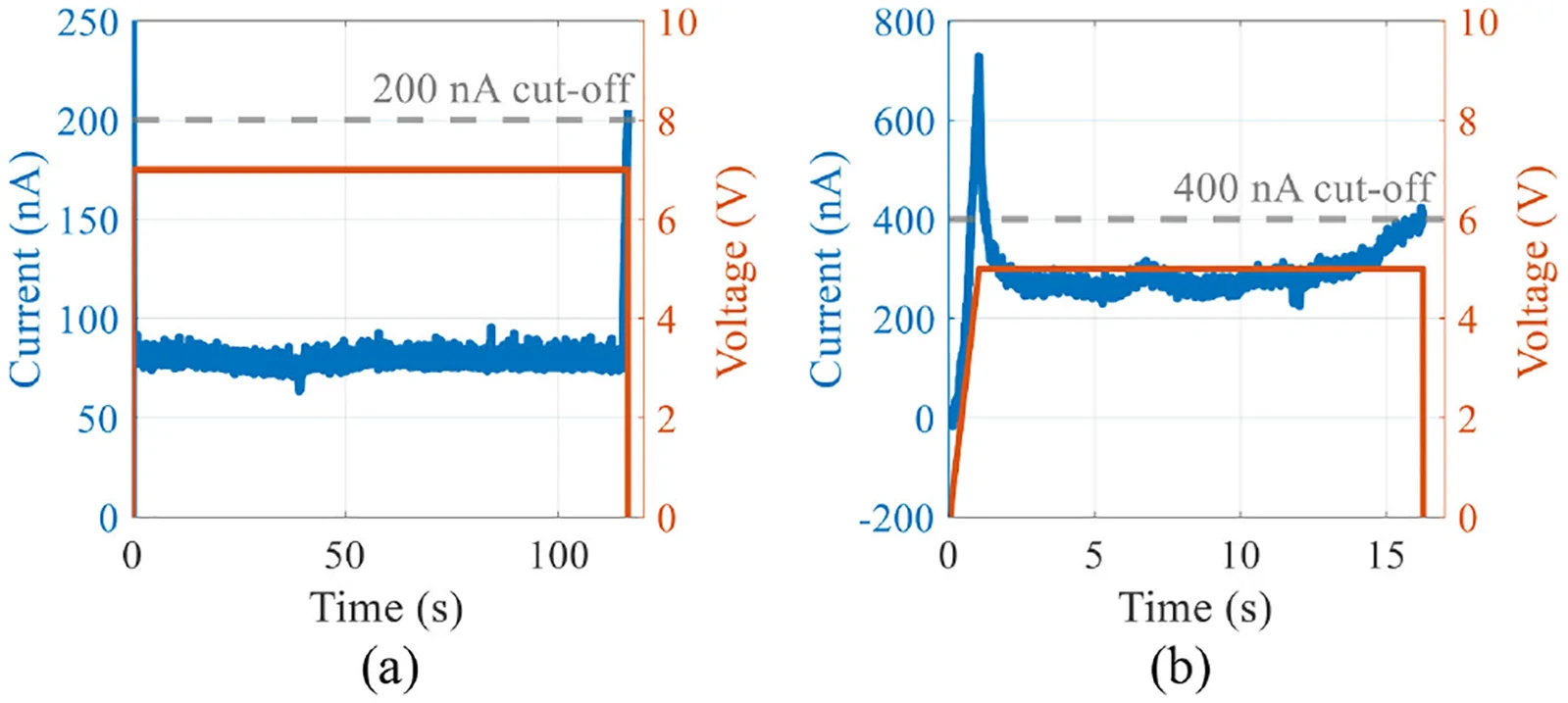
The fabrication traces of (a) 6 nm pore and (b) 10 nm pore. Step voltage profile is applied in (a), and ramping voltage profile is applied in (b).

In the step voltage mode, a constant voltage is applied instantaneously across the membrane and held for the duration of the experiment. Figure 7(a) presents a representative current trace recorded during step-voltage CT-CDB. Upon application of the voltage, a transient capacitive spike is observed, after which the leakage current settles to a stable baseline of approximately 125 nA. This steady-state current remains well below the cut-off threshold and then exceeds it, forming a ~6 nm pore (dashed line, ~200 nA). The step voltage mode is particularly useful for membranes that require prolonged stress to initiate breakdown, and provides a stable electrical environment for studying the kinetics of defect nucleation.

In the ramping voltage mode, the applied voltage increases linearly from zero to a specified target value over a user-defined ramp rate, after which it is held constant. Figure 7(b) shows a typical current trace obtained under this configuration. As the voltage ramps, the current rises and exhibits an initial transient overshoot before settling into a regime of gradual increase. The current progressively approaches and ultimately reaches the cut-off threshold corresponding to a 10 nm pore (dashed line, ~400 nA), at which point the system automatically terminates the voltage to prevent uncontrolled pore enlargement. The ramping mode offers finer control over the stress applied to the membrane, reducing the probability of abrupt, catastrophic breakdown events and thereby improving the yield of well-defined pores.

As illustrated in the GUI screenshot (Fig. 5), the control panel allows the user to configure both modes by specifying the target voltage, the ramping time (applicable only in ramping mode), and the cut-off current. During fabrication, the lower panel of the real-time display plots the current alongside a horizontal dashed threshold line, providing immediate visual feedback on the proximity of the current to the target cut-off value. The two voltage profiles are provided as selectable fabrication strategies rather than as directly benchmarked competing protocols in the present study; therefore, no statistically controlled superiority between step and ramping operation is inferred from the representative traces in Fig. 7.

### D. FABRICATION REPRODUCIBILITY AND PORE-SIZE STATISTICS

To quantitatively evaluate the reproducibility of the P.O.R.E.-based fabrication process beyond the representative traces shown in Fig. 7, repeated fabrication experiments were performed for target pore diameters of 6, 10, 15, and 20 nm. A total of 20 independent nanopore chips were tested for each target condition. The fabrication outcomes are summarized quantitatively in Table 3, including the number of successful pores, fabrication yield, final pore diameter, target-size error, initial breakdown time, and observed failure modes.

**FIGURE 8.**
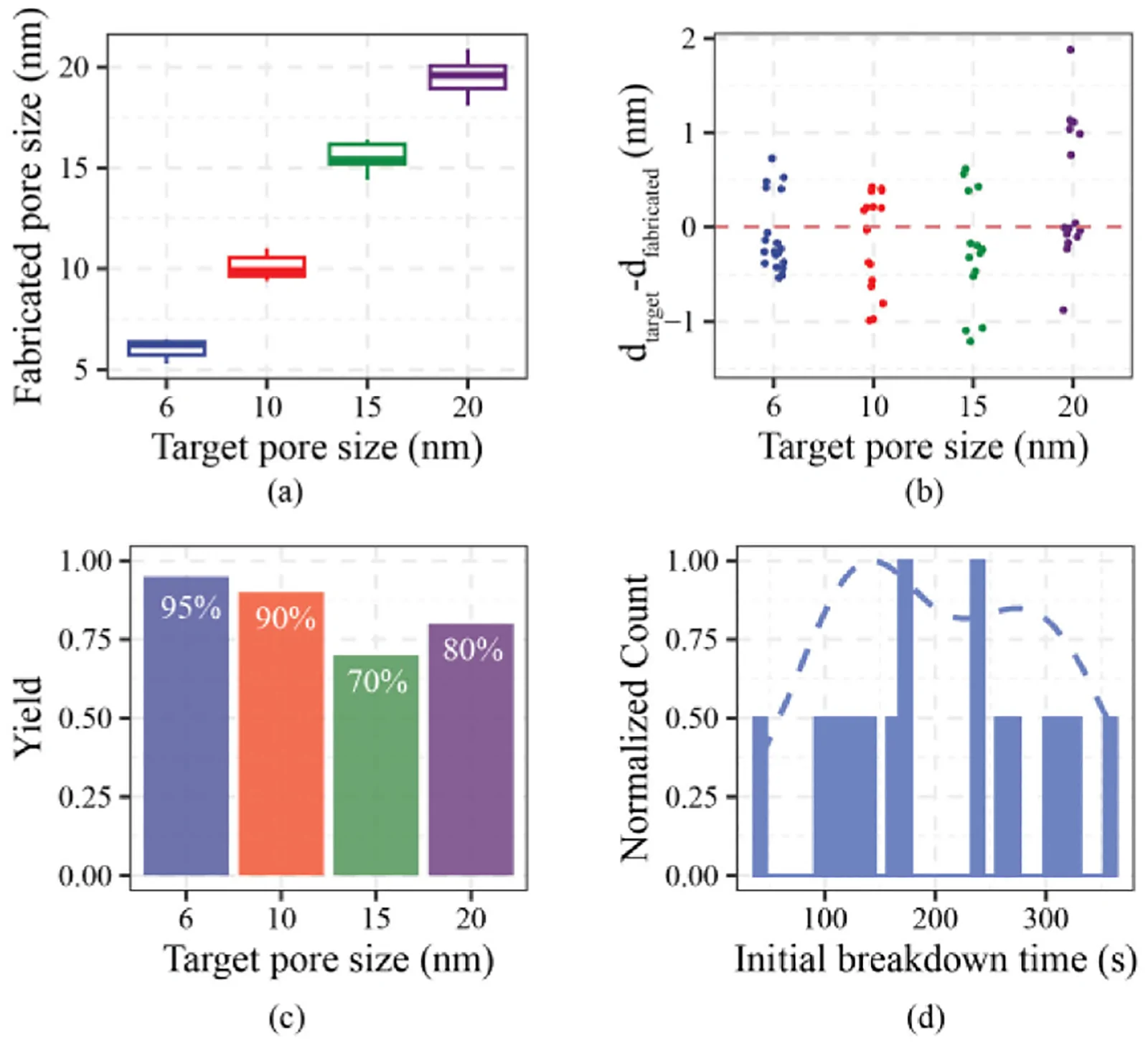
Statistical validation of nanopores fabricated using the P.O.R.E. system. (a) Final conductance-derived pore-size distributions for target diameters of 6, 10, 15, and 20 nm. Twenty independent fabrication attempts were performed for each target condition, and the box plots include successfully fabricated pores only. (b) Target-size error for the successfully fabricated pores, defined as $d_{\text{target}}-d_{\text{fabricated}}$. Positive values indicate pores smaller than the target diameter, whereas negative values indicate pores larger than the target diameter. (c) Fabrication yield calculated from all 20 fabrication attempts for each target condition. The corresponding yields were 95%, 90%, 70%, and 80% for the 6, 10, 15, and 20 nm target groups, respectively. (d) Distribution of the initial dielectric-breakdown time, defined as the interval from voltage application to detection of the first breakdown event. The initial breakdown time does not include subsequent conductance-guided pore enlargement used, when required, to reach larger final target diameters.

It should be noted that the target diameter does not necessarily correspond to the pore size generated immediately at dielectric breakdown. Initial pore nucleation during CT-CDB is stochastic, and the first breakdown event sometimes produces a relatively small pore with a variable diameter. For larger target sizes, if the initially formed pore is much smaller, it will be subsequently enlarged through additional voltage application (1V~4V) while monitoring its ionic conductance and pore size observer estimation until the desired size range is reached. Therefore, the target-size statistics reported in Table 3 and Fig. 8 refer to the final conductance-derived pore diameter after completion of the fabrication procedure, rather than the diameter immediately following initial breakdown.

Fig. 8(a) shows the final pore-size distributions of the successfully fabricated devices for the four target groups. Because only successful fabrication outcomes are included in the pore-size analysis, the numbers of samples represented in the 6, 10, 15, and 20 nm groups are 19, 18, 14, and 16, respectively. Fig. 8(b) presents the corresponding signed sizing error, defined as $d_{\text{target}}-d_{\text{fabricated}}$, where positive values indicate pores smaller than the intended target and negative values indicate pores larger than the target. The corresponding distributions demonstrate that the successfully fabricated pores remain centered near their intended target sizes, although the dispersion becomes more pronounced for the larger target diameters. This increased variation is consistent with the additional enlargement required after the stochastic initial breakdown event.

Across the 20 fabrication attempts performed for each target condition, successful fabrication yields of 95%, 90%, 70%, and 80% were obtained for target diameters of 6, 10, 15, and 20 nm, respectively, as summarized in Fig. 8(c). Here, a successful fabrication is defined as a stable Ohmic pore obtained and the final conductance-derived diameter fell within ±2 nm of the intended target without membrane rupture or uncontrolled enlargement. These two factors, as shown in Table 3 as well, are the main mechanisms of fabrication failure. Specifically, the uncontrolled post-breakdown enlargement reduces the yield observed for some of the larger target pore sizes.

Figure 8(d) further summarizes the distribution of the initial dielectric-breakdown time across the fabrication experiments. The breakdown time is defined as the interval between the onset of the fabrication voltage and detection of the first breakdown event and therefore does not include any subsequent pore-enlargement procedure. Because each fabrication attempt was performed using an independent SiN_x_ membrane chip, the distributions reported in Fig. 8 inherently include membrane-to-membrane variability rather than repeated measurements on the same device. In general, the pore formation by P.O.R.E. system needs much less than 10 minutes.

The 6–20 nm range investigated in this work should be regarded as the experimentally validated operating window of the present P.O.R.E. implementation rather than as a fundamental fabrication limit. This range provides a practical compromise between fabrication controllability and downstream sensing utility. Compared with sub-5-nm pore formation, which requires substantially tighter control of the rapid post-breakdown growth process, pores in the present range produce larger and more readily resolved conductance changes for threshold-based fabrication control. Across this range, the fabricated nanopores exhibit Ohmic I-V behavior and low-frequency noise without anomalous spectral features, while the molecular measurements further demonstrate stable open-pore baselines over 200-s recordings. From an application perspective, this size window spans dimensions relevant to a broad class of biomolecular analytes: the present study directly validates double-stranded λ-DNA using 6- and 10-nm pores and human holo-transferrin using 15- and 20-nm pores. Larger pores within this range are also compatible with the characteristic dimensions required for sensing larger proteins and nanoscale biological assemblies, including viral vectors. Thus, the 6–20 nm range represents a practically useful regime for accessible CT-CDB fabrication and biomolecular nanopore studies.

These results demonstrate that P.O.R.E. provides reproducible pore-size targeting over the experimentally validated 6–20 nm range, while the increasing spread observed for larger target diameters reflects the additional enlargement required following the stochastic initial breakdown event.

### E. PORE SIZE AND GROWTH RATE ESTIMATIONS USING THE OBSERVER

**FIGURE 9.**
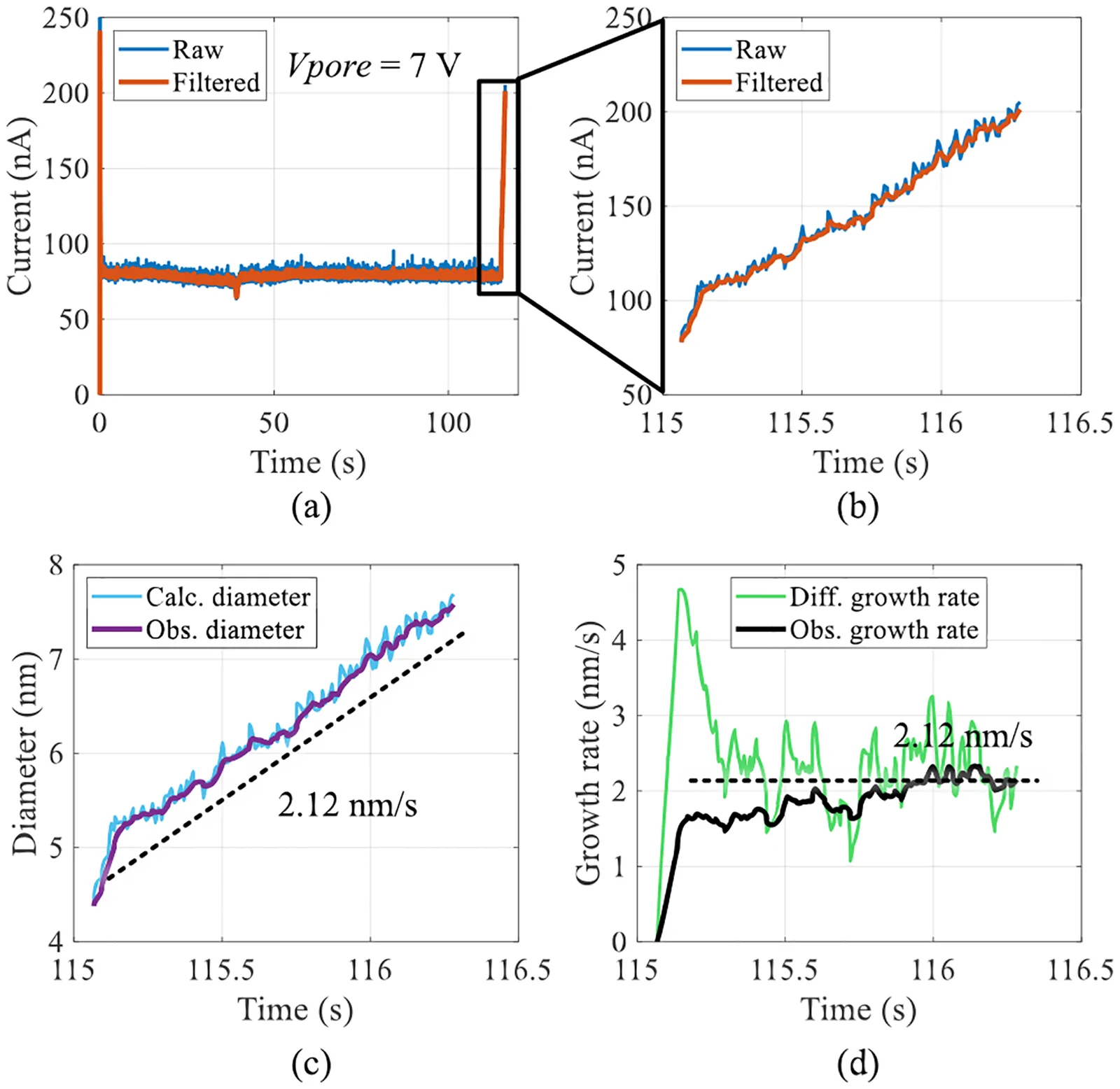
(a) Raw current from Fig. 9(a) and EMA-filtered current. (b) Open-pore current after pore fabrication. (c) Calculated and observer-estimated pore diameters. (d) Pore growth rates obtained from the calculated diameter and the observer estimate.

**FIGURE 10.**
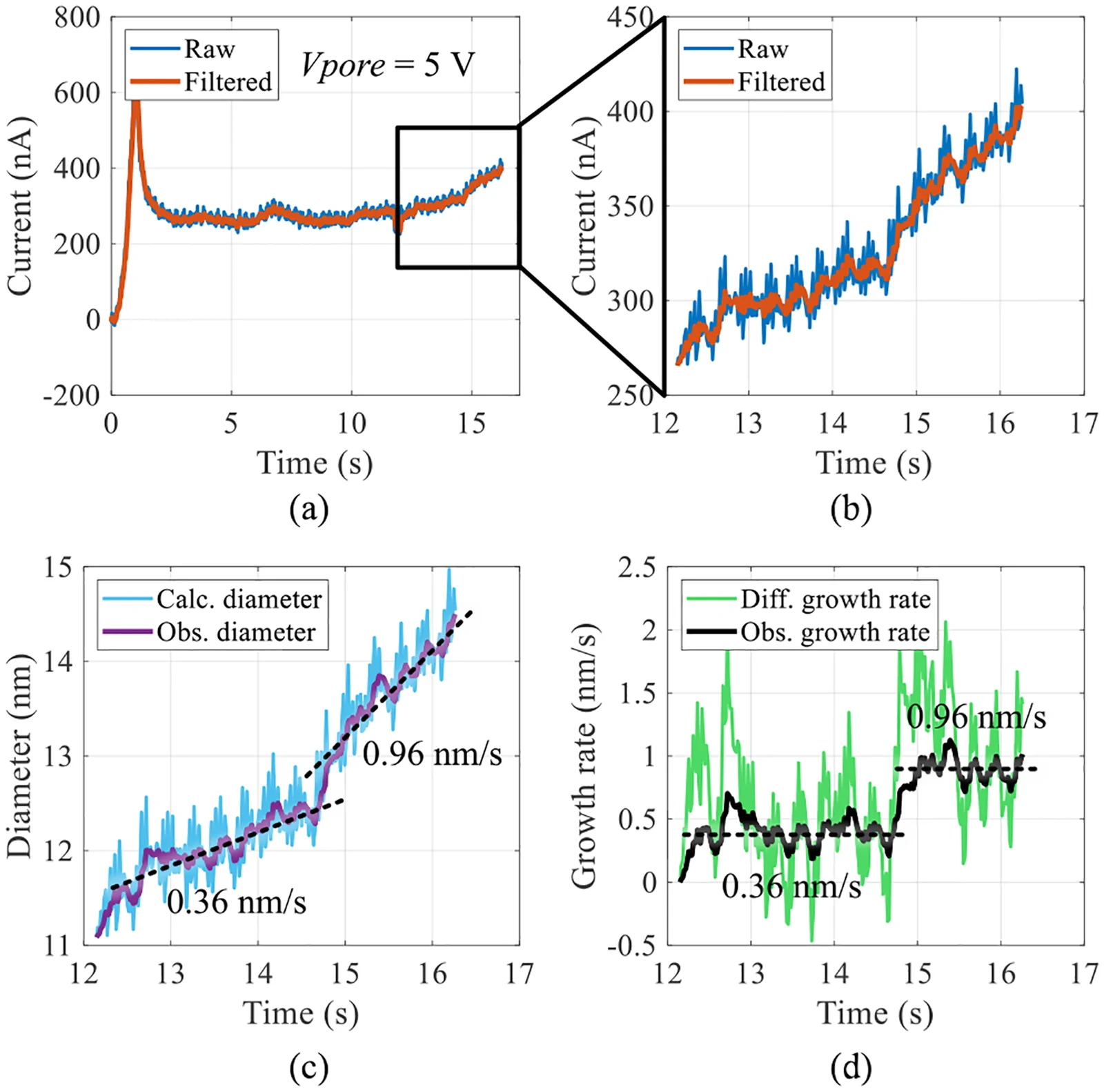
(a) Raw current from Fig. 9(b) and EMA-filtered current. (b) Open-pore current after pore fabrication. (c) Calculated and observer-estimated pore diameters. (d) Pore growth rates obtained from the calculated diameter and the observer estimate.

Real-time pore size (diameter) and growth rate estimations are performed alongside the pore formation, as shown in Figs. 9 and 10. These estimations provide critical information for nanopore fabrication because they enable prediction of the pore growth process, allowing the system and the users to determine when the target pore size is approached and to terminate fabrication before excessive pore expansion occurs.

The raw current $I_{\text{pore}}$ is first filtered using an exponential moving average (EMA) to obtain $I_{\text{filter}}$, as in (7), where γ is the smoothing factor and is empirically set to 0.35.


(7) $$I_{\text{filter}}=\gamma \times I_{\text{pore}}(k)+(1-\gamma )\times I_{\text{filter}}(k-1)$$

Figures 9(a) and 10(a) show the raw $I_{\text{pore}}$ signals from Figs. 7(a) and 7(b), along with the corresponding filtered currents $I_{\text{filter}}$. The effectiveness of the smoothing process is clearly demonstrated in Figs. 9(b) and 10(b), where the peak-to-peak current noise is reduced from 32 nA to 6 nA.

Subsequently, pore diameter $d_{\text{calc}}$ is estimated from the measured conductance G using (8), as described in [11].

(8) $$d_{\text{calc}}=\frac{G}{2K}\left(1+\sqrt{1+16\frac{K}{G\pi }L}\right)$$

where G is $I_{\text{pore}}/V_{\text{pore}},K$ is the electrolyte conductivity (11.4 S/m), and L is the membrane thickness (12 nm).

Moreover, the growth rate ${\dot{d}}_{\text{calc}}$ is obtained by directly differentiating $d_{\text{calc}}$, defined as:

(9) $${\dot{d}}_{\text{calc}}=\frac{d_{\text{calc}}(k)-d_{\text{calc}}(k-1)}{dt}$$

Conductance-based estimation is sensitive to instantaneous current fluctuations, and numerical differentiation of the pore growth rate further amplifies noise. During the early stage following dielectric breakdown, transient current variations and stochastic ion transport introduce significant uncertainty, rendering direct estimation unsuitable for stable real-time monitoring and control. As shown in Figs. 9(c)–(d) and 10(c)–(d), the estimated pore size and growth rate exhibit peak-to-peak noise of approximately 6 nm and 1 nm/s, respectively.

The second estimation approach is based on a discrete-time Luenberger observer, which enables simultaneous estimation of pore diameter and growth rate without noise amplification from numerical differentiation. The observer-based estimation is formulated using the following dynamic and measurement models:

Dynamic model:

(10) $$d_{\text{pred}}(k)=d_{\text{obs}}(k-1)+dt\times {\dot{d}}_{\text{obs}}(k-1)$$

(11) $${\dot{d}}_{\text{pred}}(k)={\dot{d}}_{\text{obs}}(k-1)$$

Measurement model:

(12) $$e(k)=d_{\text{calc}}(k)-d_{\text{pred}}(k)$$

(13) $$d_{\text{obs}}(k)=d_{\text{pred}}(k)+a\times e(k)$$

(14) $${\dot{d}}_{\text{obs}}(k)={\dot{d}}_{\text{pred}}(k)+\frac{\beta }{dt}\times e(k)$$

where the dynamic model predicts both the pore diameter $d_{\text{pred}}$ and its growth rate ${\dot{d}}_{\text{pred}}$. The measurement model then estimates $d_{\text{obs}}$ and ${\dot{d}}_{\text{obs}}$ using the error between $d_{\text{calc}}$ and $d_{\text{pred}}$, with smoothing factors a=0.6 and β=0.01 in (13) and (14). The sampling time dt is given as 12.5 ms (80 Hz).

The observer-estimated diameter $d_{\text{obs}}$ is compared in Figs. 9(c) and 10(c). The noise components present in the raw $I_{\text{pore}}$-based estimation are effectively suppressed in $d_{\text{obs}}$, where the peak-to-peak noise of $d_{\text{calc}}$ is reduced from 6 nm to 0.4 nm in $d_{\text{obs}}$. The estimated growth rates ${\dot{d}}_{\text{calc}}$ and ${\dot{d}}_{\text{obs}}$ are also compared as shown in Figs. 9(d) and 10(d). It is observed that the peak-to-peak noise of ${\dot{d}}_{\text{calc}}$ is reduced from 1 nm/s to 0.15 nm/s in ${\dot{d}}_{\text{obs}}$. Moreover, ${\dot{d}}_{\text{obs}}$ clearly captures the growth trend (slope), as shown in Figs. 9(c) and 10(c).

The smoothing parameters a and β are selected based on the Benedict–Bordner relation [18], derived for systems subject to random velocity steps and measurement noise. The optimal trade-off between transient response and noise attenuation is given by:

(15) $$\beta =\frac{a^{2}}{2-a}$$

For a∈[0.5,0.9], (15) yields β∈[0.16,0.73]. In practice, additional damping is applied to suppress overshoot and high-frequency sensitivity, resulting in gains below the theoretical optimum. In this work, a=0.6 is selected, giving β≈0.257, which is further empirically reduced to β=0.01 to ensure a heavily damped response for stable growth rate estimation.

**FIGURE 11.**
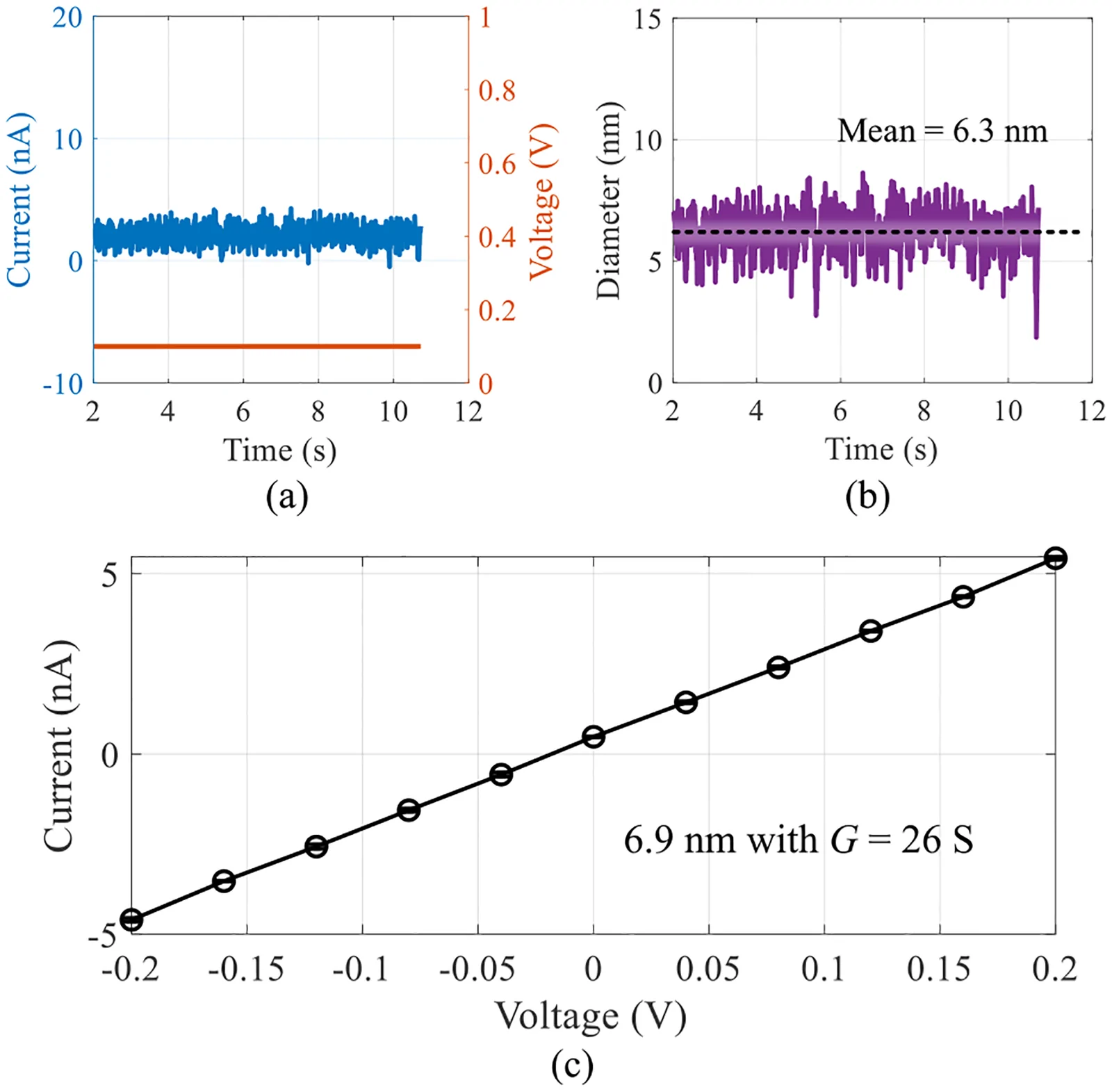
Validation of pore size estimation. (a) Pore current under 0.1 V bias. (b) Estimated pore size using the observer. (c) Estimated pore size using AxoPatch 200B I-V measurements. The estimated pore diameters are 6.3 nm (Fig. 10(b)) and 6.9 nm (Fig. 10(c)), respectively.

To verify pore size estimation accuracy, an additional nanopore chip with identical parameters in (8) is fabricated, and pore size was estimated under a 0.1 V bias using the proposed system, as shown in Fig. 11(a) and (b). The results are compared with I–V measurements obtained using an Axopatch 200B, as shown in Fig. 11(c). The estimated pore diameter from the Axopatch data is 6.9 nm, while the proposed system yields an average value of 6.3 nm, resulting in a 0.6 nm difference. This sub-nanometer discrepancy reflects numerical estimation smoothness rather than true measurement resolution, which is ultimately limited by membrane thickness uncertainty (12 ± 2 nm) and the model assumptions in (8).

To further evaluate the pore-size estimation accuracy, the validation was extended to multiple independent nanopores spanning approximately 6–20 nm. For each nanopore, the observer-estimated diameter obtained using the P.O.R.E. system under a 0.1 V bias was compared with the diameter independently derived from the slope of the Axopatch 200B I–V curve. Across the evaluated nanopores, the mean absolute error was 0.38 nm, with a standard deviation of 0.15 nm and a maximum absolute error of 0.62 nm. These results demonstrate sub-nanometer agreement between the P.O.R.E. observer and the Axopatch-derived conductance reference over the tested pore-size range.

The sensitivity to the assumed membrane thickness was also evaluated by recalculating the pore diameter using membrane thicknesses of 10, 12, and 14 nm, corresponding to the specified 12 ± 2 nm tolerance. Relative to the value obtained using 12 nm, the estimated diameter varied by approximately ±0.4 nm for a 6 nm pore, ±0.6 nm for a 10 nm pore, ±0.8 nm for a 15 nm pore, and ±1.0 nm for a 20 nm pore. Thus, membrane-thickness uncertainty has a greater influence on larger pores but remains within approximately ±1 nm over the experimentally validated range. The observer currently operates strictly as an open-loop state estimator rather than an active closed-loop controller.

These results demonstrate the effectiveness of the proposed observer for future MPC-based closed-loop nanopore fabrication. By jointly estimating pore diameter and growth rate, the method enables predictive control of breakdown dynamics and improved repeatability of target pore sizes.

### F. POST-FABRICATION ELECTRICAL CHARACTERIZATION

**FIGURE 12.**
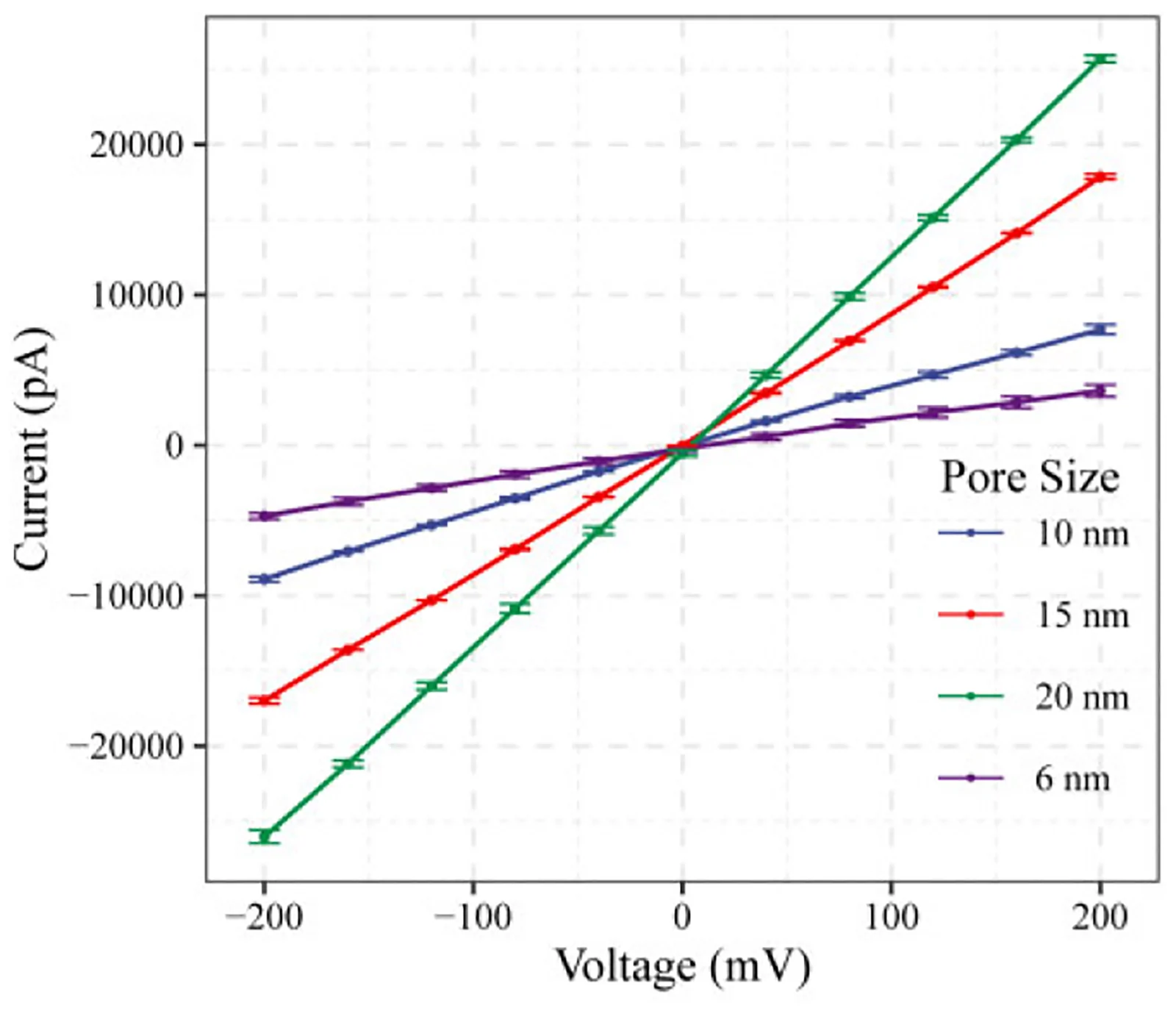
I-V curves of solid-state nanopores fabricated by P.O.R.E. system with various pore sizes from 6 nm to 20 nm.

Following CT-CDB fabrication, the nanopores were characterized by measuring their current–voltage (I-V) responses in a symmetric electrolyte configuration. Fig. 12 presents I-V curves acquired over a bias range of ±200 mV for nanopores with estimated diameters of 6, 10, 15, and 20 nm. All four types of pores exhibit linear, ohmic behavior across the entire voltage range, confirming that the fabricated apertures are geometrically well-defined and free of significant rectification artifacts that would indicate asymmetric or irregular pore geometries.

The measured conductance scales monotonically with pore diameter, consistent with the expected relationship in which the ionic conductance of a cylindrical nanopore depends on both the pore cross-sectional area and the access resistance contributions from the bulk electrolyte [17], [19]. In 1 M KCl-Tris solution with conductivity of 11.4 S/m and ~pH 8, the smallest pore (6 nm) yields a conductance of approximately 18 nS, while the largest (20 nm) reaches approximately 125 nS, spanning nearly an order of magnitude and demonstrating the ability of the P.O.R.E. system to produce nanopores across a broad and practically relevant size range. The error bars, representing the standard deviation over repeated measurements, remain small relative to the mean values, indicating good electrical measurement stability across repeated measurements.

**FIGURE 13.**
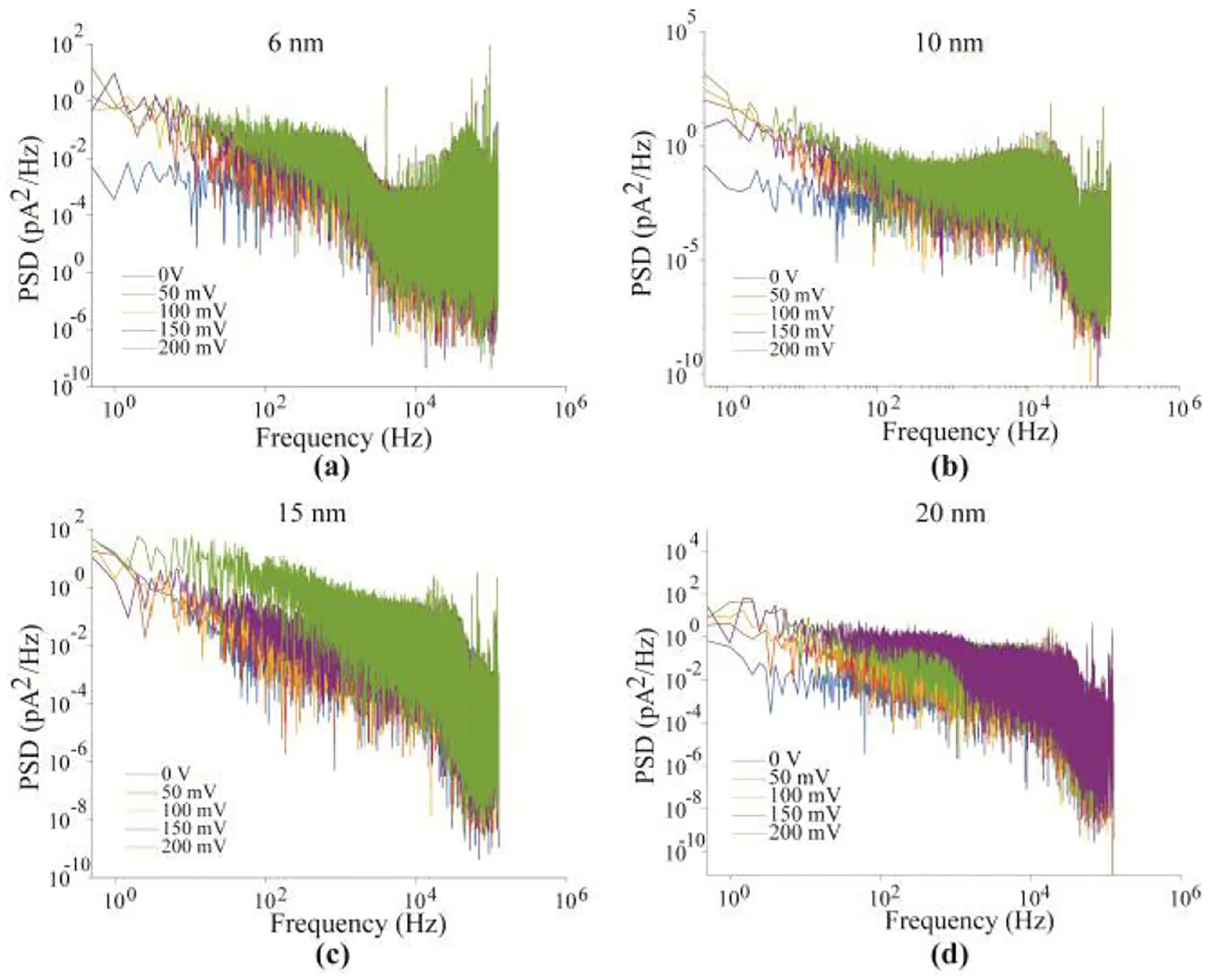
Power spectrum density (PSD) distributions of nanopores with different pore sizes. The measuring voltage range is across 0~200 mV in 1M KCl with pH~8. Low frequency magnitudes indicate the basic noise level of solid-state nanopore devices.

To further assess the quality of the fabricated nanopores, power spectral density (PSD) analysis of the ionic current noise is performed for pores of 6, 10, 15, and 20 nm in diameter under applied biases ranging from 0 to 200 mV, as shown in Figs. 13(a)–(d). 60-s open pore current traces are acquired at a sampling rate of 250 kHz with 10 kHz Bassel low-pass filter. The current time series are transformed into the frequency domain using a Fourier transform, and the resulting current-noise PSD is analyzed as a function of frequency. For all four pore sizes, the spectra acquired at zero bias represents the baseline instrument noise floor and generally exhibits the lowest PSD magnitude, confirming that the measured noise originates predominantly from the ionic transport through the pore rather than from the measurement electronics.

The low-frequency region of the spectra is of particular interest, as the 1/f noise in this regime serves as a sensitive indicator of nanopore quality [16], [20]. Flicker noise in solid-state nanopores is generally attributed to charge carrier number fluctuations at the pore surface, and its magnitude is strongly influenced by surface defects, geometric irregularities, and pore wall roughness. Across all four pore sizes, the 1/f noise component increases monotonically with the applied bias, which is consistent with the expected voltage-dependent scaling and indicates the absence of anomalous noise sources such as partial blockages, nanobubble formation, or unstable surface states. Importantly, no abrupt spectral peaks or irregular features are observed in the low-frequency regime at any bias condition, suggesting that the pores fabricated by the P.O.R.E. system are free of significant structural defects that would compromise measurement reliability.

The low-frequency PSD magnitudes do not scale proportionally with pore diameter. Rather, the low-frequency current-noise PSD spans approximately 10^−4^ ~10^3^ pA^2^/Hz, without exhibiting anomalous spectral features indicative of unstable ionic transport or unusually high low-frequency noise [9]. These electrical characteristics, with the fabrication statistics reported above, support the ability of the P.O.R.E. system to reproducibly produce electrically stable nanopores across the investigated 6–20 nm range. This, along with the 12.5 ms total feedback latency resulting from the 80 Hz polling cycle, ensures that the lower voltage regime (7–9 V) can reliably halt pore growth to produce stable pores.

## V. FUNCTIONAL VALIDATION OF P.O.R.E.-FABRICATED NANOPORES BY SINGLE-MOLECULE SENSING

To evaluate the functional quality of nanopores fabricated using the P.O.R.E. platform, single-molecule translocation measurements were performed using λ-phage DNA (λDNA, ~48.5 kbp) and human holo serum transferring (hSTf, ~80 kDa) as representative nucleic-acid and protein analytes, respectively. It should be emphasized that the P.O.R.E. onboard measurement chain is designed primarily for dielectric-breakdown monitoring and feedback-controlled nanopore fabrication rather than high-bandwidth translocation recording. Therefore, the single-molecule ionic-current traces presented in this section were acquired using a dedicated low-noise nanopore recording system consisting of an Axopatch 200B patch-clamp amplifier and a Digidata 1440A data-acquisition unit (Molecular Devices Inc.). These experiments are intended to validate the sensing functionality and electrical quality of the nanopores fabricated by P.O.R.E., rather than to demonstrate high-bandwidth single-molecule acquisition using the P.O.R.E. electronics themselves.

For single-molecule measurements, the nanopore chip was mounted in a fluidic cell containing 1 M KCl with 10 mM Tris, pH~8. Ionic current was recorded using Axopatch 200B under an applied transmembrane bias of 150 mV for λDNA and 100 mV for hSTf. The current signal was sampled at 250 kHz and low-pass filtered at 10 kHz. The final analyte concentrations were 40 pM for λDNA and 5 nM for hSTf. One independently fabricated nanopore is used for each pore-size/analyte condition. For λDNA, 217 and 222 events were detected in the 6 and 10 nm pores, respectively, corresponding to event rates of 0.70 and 0.74 s^−1^. For hSTf, 2216 and 2143 events were detected in the 15 and 20 nm pores, respectively, with corresponding event rates of 7.30 and 7.14 s^−1^.

**FIGURE 14.**
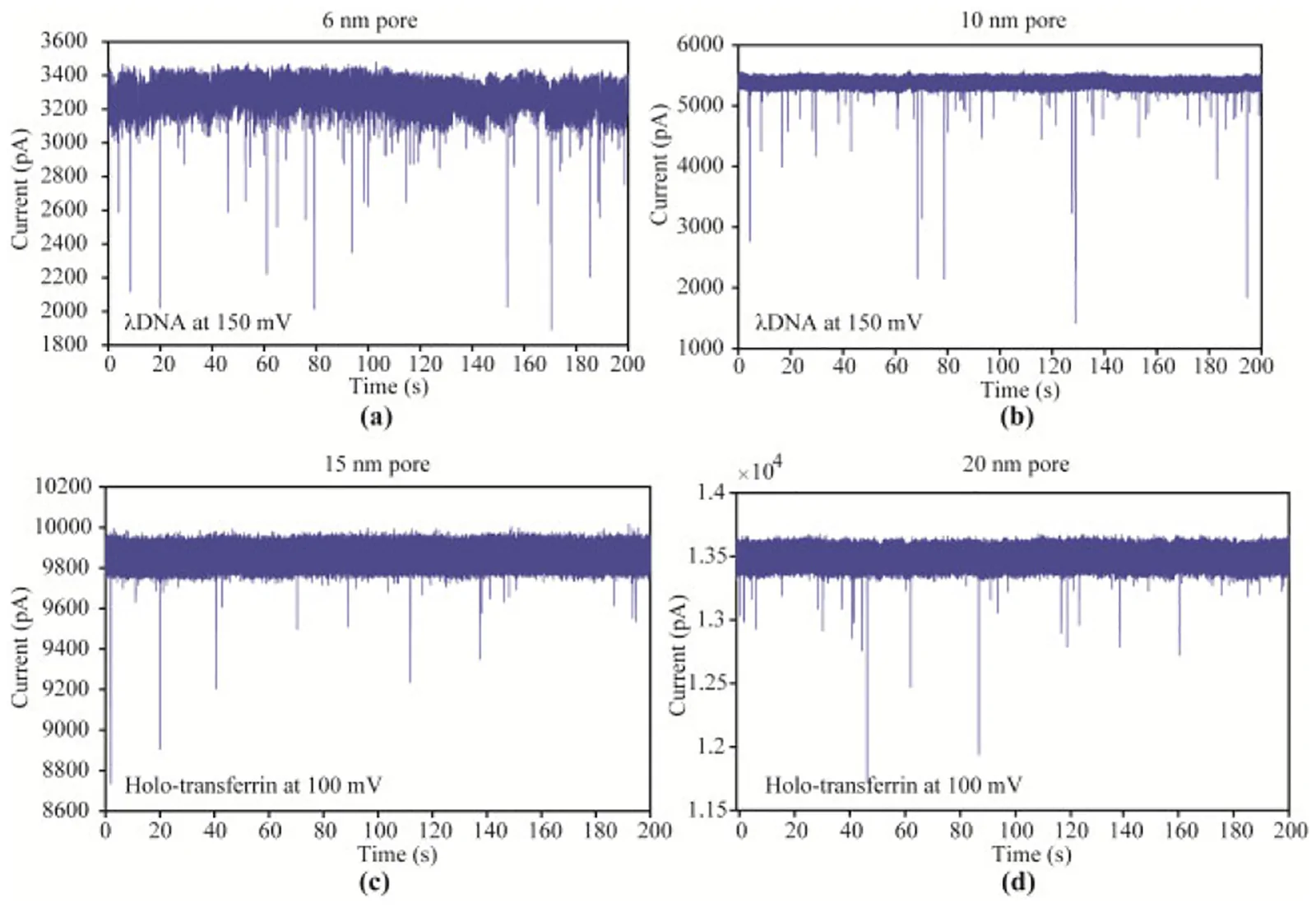
Bio-molecule translocation traces in 200 seconds from solid-state nanopores with various pore sizes (6 nm, 10 nm, 15 nm and 20 nm). All pores are fabricated by P.O.R.E. system. (a) and (b) are λDNA translocations at 150 mV. (c) and (d) are human holo serum transferrin (hSTf) translocations at 100 mV.

Fig. 14 presents 200-second continuous current traces recorded from nanopores of four different diameters using the external low-noise amplifier described above. For λDNA translocation experiments, pores of 6 nm and 10 nm were employed under a transmembrane bias of 150 mV (Fig. 14(a) and (b)), while for hSTf detection, pores of 15 nm and 20 nm were used at 100 mV (Fig. 14(c) and (d)). The open-pore current remains stable over the 200-s recording interval for all four tested pore conditions, supporting the short-term electrical stability of the fabricated nanopores during translocation measurements, and further corroborating the structural and electrical stability of the P.O.R.E. fabricated pores demonstrated in the noise analysis.

In the λDNA experiments, the 6 nm pore (Fig. 14(a)) exhibits frequent downward current transients with large blockade amplitudes, consistent with the tight geometric confinement of the double-stranded DNA molecule (~2.2 nm in cross-section) within the pore lumen [21]. The 10 nm pore (Fig. 14(b)) records translocation events with comparatively smaller ionic blockades, as expected from the reduced volume exclusion ratio when the pore diameter significantly exceeds the molecular cross-section. For hSTf translocation, the 15 nm pore (Fig. 14(c)) produces clearly resolved blockade events with appreciable current reductions, while the 20 nm pore (Fig. 14(d)) yields shallower transients that remain distinguishable from the baseline noise. The observation that the blockade depth decreases systematically as the pore-to-analyte size ratio increases is consistent with volumetric exclusion models and confirms that the detected events originate from genuine molecular translocations rather than noise signals [22].

Translocation events are extracted and analyzed using EventPro, a MATLAB-based nanopore data-analysis platform previously developed for baseline construction, event extraction, and abrupt change-based multilevel fitting [23]. The Gaussian smoothing baseline-fitting mode in EventPro is used for all traces, with identical analysis settings applied within each analyte group. For each detected event, the open-pore current I_0_, blockade current ΔI, relative current drop $\Delta \text{I}/{\text{I}}_{0}$ and dwell time are obtained and calculated for subsequent statistical analysis.

**FIGURE 15.**
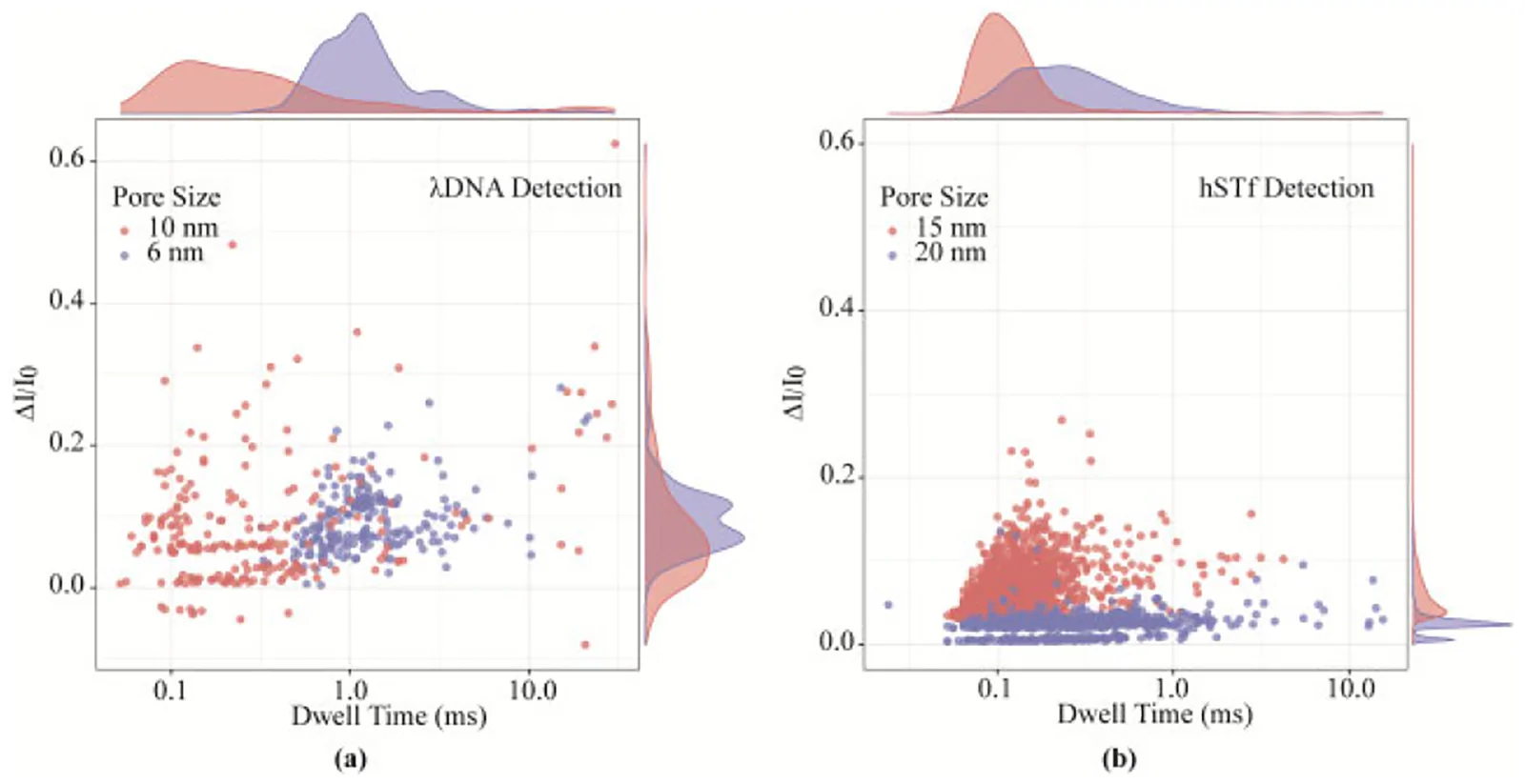
Scattered distributions between dwell time and relative current blockade ($\Delta \text{I}/{\text{I}}_{0}$). (a) λDNA translocation distributions in 6 and 10 nm pores. (b) Human holo serum transferrin translocation distributions in 15 and 20 nm pores. The dwell time axes from both graphs are demonstrated by logarithmic coordinates. The density distributions of two parameters are also shown on top and right sides.

To further characterize the translocation dynamics, the dwell time and relative current blockade ($\Delta \text{I}/{\text{I}}_{0}$) of individual events were extracted and plotted as two-dimensional scatter distributions with accompanying marginal density histograms, as shown in Fig. 15.

For λDNA translocation (Fig. 15(a)), events recorded from the 6 nm pore cluster at longer dwell times and higher $\Delta \text{I}/{\text{I}}_{0}$ values compared to those from the 10 nm pore. The median $\Delta \text{I}/{\text{I}}_{0}$ values were 0.088 and 0.062, while the corresponding median dwell times were 1.152 ms and 0.236 ms for the 6 and 10 nm pores, respectively. The elevated ionic blockade in the smaller pore reflects the greater volumetric occupancy of the DNA strand within the confined aperture, while the longer dwell time is attributed to the enhanced interaction between the molecule and the pore walls at tighter confinements, which increases the effective friction opposing electrophoretically driven transport. The dwell time distribution of the 10 nm pore is shifted toward shorter values, consistent with the reduced steric hindrance in a larger channel. Notably, the 10 nm pore also exhibits a broader spread in $\Delta \text{I}/{\text{I}}_{0}$, which may arise from folded DNA configurations entering the pore in multi-file conformations, a phenomenon that is geometrically suppressed in the 6 nm pore where only single-file threading is sterically permitted [3].

For hSTf translocation (Fig. 15(b)), the median $\Delta \text{I}/{\text{I}}_{0}$ values were 0.051 and 0.024 for the 15 and 20 nm pores, respectively, with median dwell times of 0.108 ms and 0.240 ms. Events from the 15 nm pore display higher $\Delta \text{I}/{\text{I}}_{0}$ values and a more compact dwell time distribution, whereas the 20 nm pore produces events with lower ionic blockades and a broader spread in dwell time. The hydrodynamic diameter of hSTf is approximately 7~8 nm, and the larger pore-to-analyte ratio in the 20 nm case results in a reduced volume exclusion fraction and a correspondingly weaker current blockade signal [24]. Despite the smaller signal amplitude, the events remain well-resolved from the baseline, confirming that the 20 nm pore retains sufficient sensitivity for protein detection.

These translocation measurements confirm that nanopores fabricated by the P.O.R.E. platform across the investigated 6–20 nm range are suitable for downstream single-molecule sensing of both nucleic-acid and protein analytes. Importantly, this validation reflects the sensing quality of the fabricated nanopores, while high-bandwidth current acquisition in the present study was performed using a dedicated external low-noise amplifier.

## VI. CONCLUSION

In this work, we demonstrated that a fully functional solid-state nanopore fabrication platform can be built from commodity components for under $100 USD (enclosure and flow-cell cartridge, experimental consumables excluded) without sacrificing electrical precision or pore quality (Table 4). Its broader significance lies in lowering the field’s high entry barriers, specifically the capital cost of beam-based milling and the recurring license costs or rigidity of proprietary control software like MATLAB. By consolidating fabrication and monitoring into a single USB-powered, smartphone-sized unit governed by an open, modular Python/PyQt GUI, P.O.R.E. democratizes CT-CDB fabrication, making it highly reproducible in resource-limited and educational settings. The validated 6–20 nm pore size range owns 70–95% fabrication yield with sub-nanometer mean errors, producing stable pores suitable for biomolecular sensing. However, this 6-nm lower limit is restricted by our 0.05 nA/LSB current resolution, reactive manual cut-off logic delays, and high breakdown voltages (7–9 V) of the 12 nm SiNx membrane, whereas sub-2-nm control demands 0.5–1 pA/LSB precision to isolate initial nucleation from noise.

Several limitations bound the present results. P.O.R.E. is currently a single-channel tool without parallel capability, and its measurement chain (0.05 nA/LSB) requires a separate amplifier for high-bandwidth sensing. This resolution also restricts our lower fabrication limit to ~6 nm, whereas sub-2-nm control demands 0.5–1 pA/LSB precision to isolate initial nucleation from noise. Additionally, the observer currently only estimates pore states rather than driving the control loop. This reactive, manual cut-off logic introduces delays that allow stochastic growth to overshoot, which is further exacerbated by high breakdown voltages (7–9 V) of the 12 nm SiNx membrane where the abrupt discharge of accumulated capacitive energy inherently enlarges the initial pore. Consequently, our immediate future priority is to close the control loop around the observer. By utilizing its real-time diameter and growth-rate estimations—which suppress diameter noise from 6 nm to 0.4 nm and growth-rate noise from 1 nm/s to 0.15 nm/s—within a model-predictive control framework, the system can anticipate threshold crossings and dynamically modulate voltage. This will replace the current manual cut-off heuristics with automated, model-based size targeting to eliminate overshoot, tighten size distributions, and achieve reliable sub-5-nm precision.

A second direction is extending single-channel operation to genuinely parallel, many-channel fabrication. Scaling to an N-channel array with per-channel current sensing and a dedicated observer per pore would turn the platform from a serial tool into a high-throughput engine. The already-decoupled Device and Control modules are well suited to managing concurrent control threads, though inter-channel crosstalk, electrode multiplexing, and per-channel calibration must be addressed. Complementary efforts include a faster, lower-noise front-end so that fabrication and high-bandwidth sensing can coexist on one device, broadening the protocol library to other membrane materials and pulsed breakdown schemes, and exploiting the logged datasets for machine-learning-assisted process control.

These developments provide a pathway from the present accessible single-channel device toward future autonomous and parallel solid-state nanopore fabrication systems. More broadly, they illustrate how low-cost and extensible instrumentation can lower the technical and financial barriers to nanopore fabrication and potentially facilitate broader access to solid-state nanopore technology for research and single-molecule sensing applications.

## Figures and Tables

**TABLE 1.** Cost analysis of the PCB in this work.

Description	Value	Quantity	Cost ($)
Microcontroller	Arduino UNO R3	1	27.6
DAC	MCP4725	1	4.95
ADC	ADS1115	1	14.95
Op-amp	AD820	3	21.87
Power supply	NMA0515SC	1	5.05
Passive components	R,L,C *	—	1.3
PCB board	—	1	10
Total			85.72

“—” indicates that the corresponding value or quantity varies with the design specifications.

“*” indicates resistors (R), inductors (L) and capacitors (C).

**TABLE 2.** Control parameters of P.O.R.E. system.

Parameter	Unit	Description	Reference Value
Target Voltage	V	The applied voltage across membrane	4 – 9
Voltage Mode	—	How the voltage increases to the target level	Stage/Ramping
Ramping Time	s	Time set for ramping voltage	1
Cut-off Current	nA	The fabrication threshold level (refer to leakage current level)	1.2× – 2.5×

**TABLE 3.** Statistical summary of P.O.R.E-based nanopore fabrication.

Target pore size (nm)	Total attempts	Successful pores*	Yield	Final pore size (nm)	Mean absolute error (nm)	Main failure mode	Initial breakdown time (s)**
6	20	19	95%	6.06±0.42	0.38	Rupture	
10	20	18	90%	10.10±0.54	0.46	Rupture	
15	20	14	70%	15.52±0.69	0.72	Enlargement failed	202.76±93.49
20	20	16	80%	19.53±0.82	0.71	Enlargement failed	

* Include successfully enlarged pores to the target pore size range.

** Overall initial breakdown time: 202.76 ± 93.49 s across all fabrication attempts

Note: Final pore-size statistics and Mean Absolute Error are calculated using successfully fabricated pores only. Fabrication yield is calculated using all 20 independent fabrication attempts per target condition. Initial breakdown time refers to the interval before the first dielectric-breakdown event and excludes any subsequent pore-enlargement procedure.

**TABLE 4.** Comparison of P.O.R.E. with representative recent controlled-breakdown (CBD) fabrication platforms.

Feature	P.O.R.E. (This work)	Waugh et al [11]	Cui et al. [12]	Bandara et al [13]
Platform	Arduino UNO R3, USB	Benchtop instrumentation	Microcontroller, USB	Arduino, USB
Reported cost (USD)	≈ 86; <100	N/R	N/R	<150
Control-board size	58 × 36 mm	N/R	N/R (device 170 × 100 × 55 mm)	58 × 107 mm
Components	SMD	N/R	N/R	Through-hole (DIP)
Software environment	Python / PyQt	Custom (provided with protocol)	Custom automation (closed)	MATLAB (proprietary)
Feedback / control method	Current cut-off (CT-CDB) + observer-based size estimation	Automated CDB	Bipolar voltage + Slope/Jerk + PID	Current cut-off
Demonstrated pore-size range	6–20 nm	≥ 2 nm (tunable)	2–20 nm (±0.5 nm)	2.6–12.6 nm
Real-time pore-size estimation	Yes (Luenberger observer)	No	Size control via PID (no state estimator)	No
Fabrication statistics	80 attempts; 67 successful pores	Yield >85%	N/R	60 pores
Reported yield	70–95% depending on target size	85% yield	±0.5% nm size accuracy	~80% within 1 nm of target
Target-size error / variation	MAE = 0.38–0.72 nm; SD = 0.42–0.82 nm	N/R	±0.5 nm	~80% within ±1 nm
Demonstrated sensing	DNA + protein	DNA	DNA	DNA

N/R: not reported
